# Integrated genome and transcriptome analyses reveal the mechanism of genome instability in ataxia with oculomotor apraxia 2

**DOI:** 10.1073/pnas.2114314119

**Published:** 2022-01-18

**Authors:** Radhakrishnan Kanagaraj, Richard Mitter, Theodoros Kantidakis, Matthew M. Edwards, Anaid Benitez, Probir Chakravarty, Beiyuan Fu, Olivier Becherel, Fengtang Yang, Martin F. Lavin, Amnon Koren, Aengus Stewart, Stephen C. West

**Affiliations:** ^a^DNA Recombination and Repair Laboratory, The Francis Crick Institute, London NW1 1AT, United Kingdom;; ^b^Bioinformatics and Biostatistics, The Francis Crick Institute, London NW1 1AT, United Kingdom;; ^c^Aston Medical School, Aston University, Birmingham B4 7ET, United Kingdom;; ^d^Department of Molecular Biology and Genetics, Cornell University, Ithaca, NY 14853;; ^e^Wellcome Sanger Institute, Wellcome Trust Genome Campus, Cambridge CB10 1SA, United Kingdom;; ^f^Center for Clinical Research, University of Queensland, Herston, QLD 4029, Australia

**Keywords:** DNA repair senataxin, ataxia with oculomotor apraxia, transcription stress

## Abstract

Mutations in the *SETX* gene, which encodes Senataxin, are associated with the progressive neurodegenerative diseases ataxia with oculomotor apraxia 2 (AOA2) and amyotrophic lateral sclerosis 4 (ALS4). To identify the causal defect in AOA2, patient-derived cells and *SETX* knockouts (human and mouse) were analyzed using integrated genomic and transcriptomic approaches. A genome-wide increase in chromosome instability (gains and losses) within genes and at chromosome fragile sites was observed, resulting in changes to gene-expression profiles. Transcription stress near promoters correlated with high GCskew and the accumulation of R-loops at promoter-proximal regions, which localized with chromosomal regions where gains and losses were observed. In the absence of Senataxin, the Cockayne syndrome protein CSB was required for the recruitment of the transcription-coupled repair endonucleases (XPG and XPF) and RAD52 recombination protein to target and resolve transcription bubbles containing R-loops, leading to genomic instability. These results show that transcription stress is an important contributor to *SETX* mutation-associated chromosome fragility and AOA2.

## Significance

Ataxia with oculomotor apraxia (AOA) is a progressive neurodegenerative disease characterized by early‐onset autosomal recessive cerebellar ataxia with oculomotor apraxia, peripheral axonal neuropathy, and impaired motor functions. The AOA-2 subgroup results from mutations in an RNA/DNA helicase, Senataxin, which is encoded by the *SETX* gene. Here, we carried out integrated genome and transcriptome analyses of cell lines derived from individuals with AOA2, as well as CRISPR/Cas9 generated *SETX* knockouts, and observed genome-wide chromosome fragility. Genome instability was caused by increased transcription stress and the accumulation of RNA/DNA hybrids near gene promotors, resulting in aberrant DNA repair that led to changes in gene-expression profiles. The results indicate that *SETX*-defective cells exhibit transcription stress that leads to chromosome fragility.

Transcription has been linked to mutagenesis, DNA breakage, and genomic instability. Recent studies have highlighted the consequences of transcription-replication conflicts and the formation of transcription-linked R-loops as sources of genomic instability in both prokaryotes and eukaryotes (1). R-loops are three-stranded nucleic acid structures containing an RNA/DNA hybrid and an unpaired single-strand of DNA. They are found near gene promoters and terminators, rDNA repeats, tRNA genes, DNA double-strand breaks (DSBs), replication origins, and immunoglobulin class-switch regions.

R-loops are thought to have physiological functions, which include regulating gene expression, facilitating transcription termination, and promoting class-switch recombination (2–5). However, aberrant R-loop formation and improper processing of these structures also contributes to hypermutation, DSB formation, and chromosome rearrangements, which are all sources of genomic instability and human disease (3, 6, 7). The proper regulation of R-loop homeostasis is therefore vital for the maintenance of genome integrity.

Eukaryotic cells have evolved multiple mechanisms to control R-loop formation. Unscheduled or unwanted R-loops are either degraded by the ribonucleases RNaseH1 and RNaseH2, or removed by RNA/DNA helicases, such as Senataxin (Sen1 in yeast), Aquarius, or UAP56 (8–13). Senataxin (SETX) was first identified due to its association with an inherited autosomal recessive adolescent onset disorder known as ataxia with oculomotor apraxia 2 (AOA2) (14). Mutations in the *SETX* gene are also linked to a rare, dominantly inherited, form of motor neuron disease, amyotrophic lateral sclerosis 4 (ALS4) (15). *SETX* mutations associated with AOA2 and ALS4 are generally considered to be loss-of-function and gain-of-function, respectively. AOA2 is characterized by cerebellar atrophy, early loss of reflexes, late peripheral neuropathy, oculomotor apraxia, and impaired motor functions (16). Patient-derived AOA2 cells are sensitive to DNA damaging agents, including H_2_O_2_ (17–19). AOA2 cells exhibit altered gene expression (including neuronal genes) and increased R-loop levels (20). Although a *Setx* knockout (KO) mouse has been generated, it fails to exhibit the neurodegenerative features typical of afflicted individuals (21). However, the male mice were infertile and SETX was shown to be essential for the removal of R-loops during meiotic recombination in spermatocytes.

Senataxin has been implicated in the resolution of R-loops that form during transcription regulation (22), transcription termination (10, 23–25), replication-transcription collisions (26, 27), DNA damage (28–30), meiotic gene silencing (31), and the antiviral transcriptional response (32). However, the precise molecular functions of *SETX*, and how mutations in this gene lead to AOA2 neuropathy, remain largely unknown.

In this study, we provide a genome-wide analysis of cells derived from AOA2 patients and *SETX* KOs (human and mouse). Using a variety of genomic and transcriptomic methods, we show that loss of SETX leads to a genome-wide increase in RNA polymerase II (RNAPII) levels via RNAPII pausing/stalling (transcription stress) and chromosome instability across genes and at fragile sites. Importantly, transcription stress near promoters correlated with high GCskew (strand asymmetry in the distribution of guanines and cytosines) and R-loop accumulation at promoter-proximal regions. In the absence of SETX, R-loops near gene promoters are targeted and repaired by the XPG/XPF nucleases and RAD52 recombination protein, which requires the presence of the transcription-coupled repair (TCR) factor Cockayne syndrome B (CSB). These aberrant repair reactions lead to elevated levels of DNA damage and genomic instability.

## Results

### AOA2 Cells Exhibit Transcription-Dependent Genome Instability.

**Fig. 1.**
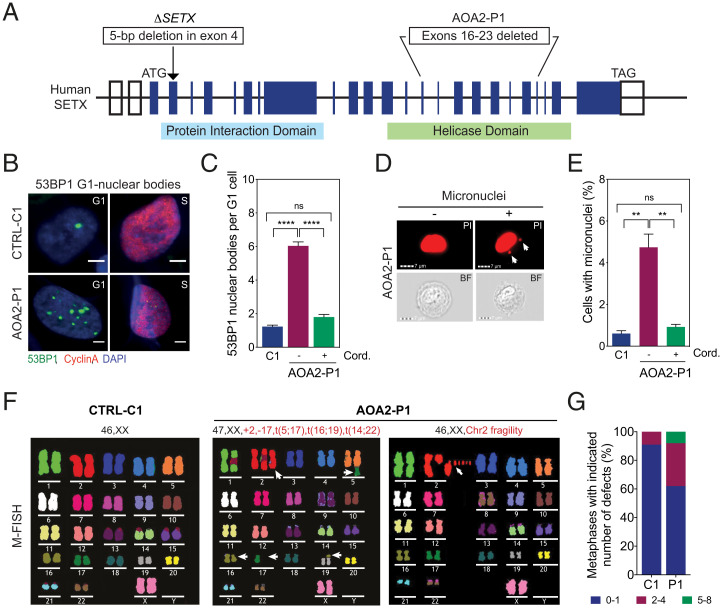
SETX deficiency promotes chromosome fragility. (*A*) Diagram of human *SETX* showing Δ*SETX* and the AOA2-P1 mutant fibroblasts. Boxes represent exons and the positions of start (ATG) and stop (TAG) codons. (*B*) Immunostaining with 53BP1 (green) and cyclin A (red) antibodies in control (CTRL-C1) and AOA2-P1 fibroblasts. DNA was stained with DAPI (blue). Representative images of G1- and S-phase cells are shown. (Scale bars, 5 μm.) (*C*) Quantification of 53BP1 NBs in G1 cells, as in *B*, with or without cordycepin. Data represent the mean ± SEM of three independent experiments with >300 cells per condition. (*D*) Imagestream imaging flow cytometry of AOA2-P1 fibroblasts. Nuclei were stained with PI. Representative PI and bright field (BF) images of mononucleated cells with and without micronuclei are shown. White arrows denote micronuclei. (Scale bars, 7 μm.) (*E*) Quantification of cells with micronuclei, as in *D*. Cells were treated with or without cordycepin. Data represent the mean ± SEM of three independent experiments, >4,000 cells per condition. (*F*) M-FISH analyses of metaphase spreads from CTRL-C1 and two AOA2-P1 fibroblasts showing deletions/amplifications and chromosome fragility. Representative karyotypes are shown. White arrows, chromosome aberrations. (*G*) Quantification of metaphases with indicated number of aberrations, as in *F*. Thirty metaphases were analyzed per condition. **P < 0.01 and ****P < 0.0001 by Mann–Whitney *U* test. *P* ≥ 0.05 is considered not significant (ns).

To investigate the genome-wide chromosome instability/fragility phenotypes associated with SETX-deficiency, we analyzed an AOA2 fibroblast cell line (designated AOA2-P1) that has a large deletion (exons 16 to 23) in the helicase domain of SETX (Fig. 1*A*) (19). Immunostaining for the DNA damage-response protein 53BP1 revealed a fourfold increase in the number of 53BP1 nuclear bodies (NBs) in cyclin A-negative G1 cells compared to control (CTRL-C1) fibroblasts, which was suppressed by treatment with the transcription elongation inhibitor cordycepin (Fig. 1 *B* and *C*). The AOA2-P1 cells also showed an approximately fivefold increase in the formation of micronuclei compared with control cells, which was again suppressed by cordycepin treatment (Fig. 1 *D* and *E*). The 53BP1 NB formation and micronuclei were also visualized by live-cell imaging of U2OS cells that stably expressed GFP-tagged 53BP1, following treatment with control (Movie S1) or *SETX* siRNA (Movie S2).

Analysis of AOA2-P1 fibroblasts by multiplex fluorescence in situ hybridization (M-FISH) and DAPI-banding revealed extensive chromosomal rearrangements (deletions, amplifications, and translocations) and numerical aberrations (Fig. 1*F* and *SI Appendix*, Fig. S1*A*). Indeed, 38% of the AOA2-P1 cells exhibited two or more aberrations compared to 9% in control cells (Fig. 1*G*). Similar results were observed with human HAP1-*SETX* KO cells (Δ*SETX*) made by CRISPR/Cas9-mediated gene targeting (*SI Appendix*, Fig. S1 *B–G*). These results show that loss or inactivation of SETX leads to transcription-dependent genomic instability.

### SETX Deficiency Induces Genome-Wide Copy Number Changes.

**Fig. 2.**
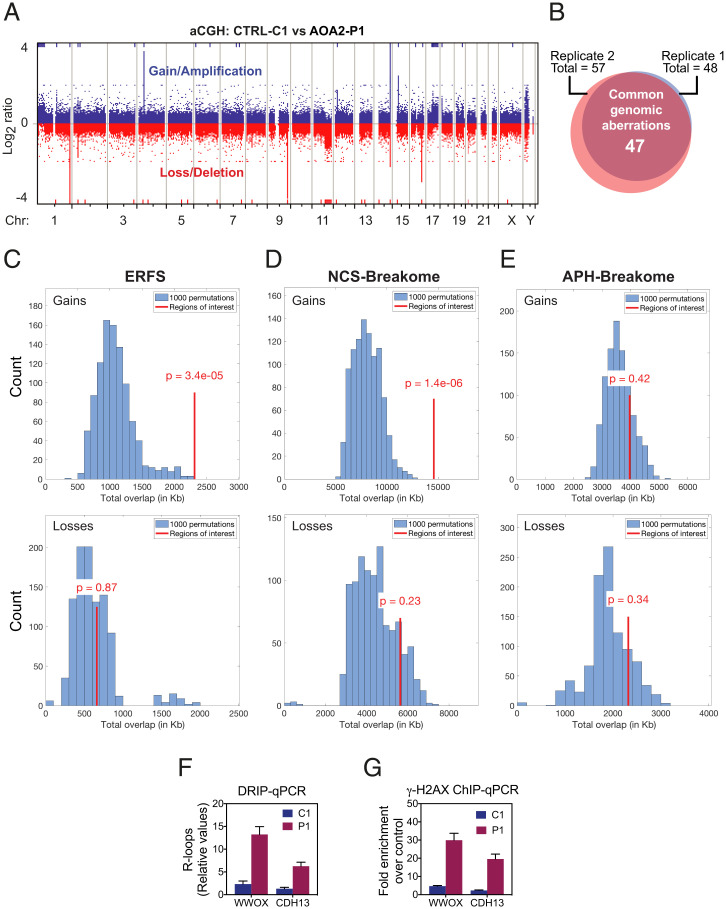
Genome-wide chromosome instability in AOA2 fibroblasts. (*A*) aCGH analyses carried out with genomic DNA from CTRL-C1 and AOA2-P1 fibroblasts. Representative whole-genome plot of aCGH profile is shown. Blue and red lines indicate chromosome regions with gains/amplifications and losses/deletions, respectively. (*B*) Venn diagram showing the overlap of chromosome aberrations detected in two independent aCGH experiments, as in *A*. (*C*, *Upper*) Histogram showing overlaps between 1,000 permuted AOA2-P1 fibroblast gain regions with ERFS. (*Lower*) Overlaps between 1,000 permuted AOA2-P1 fibroblast loss regions with the ERFSs. Red line indicates the degree of overlap (in kilobases). *P* values for the overlap compared to permutations are shown, with *P* < 0.05 considered a significant enrichment/depletion. (*D* and *E*) As *C*, showing overlaps between AOA2-P1 fibroblast gain and loss regions with NCS-breakome and APH-breakome sensitive regions. (*F*) DRIP-qPCR analyses, using the R-loop–specific S9.6 monoclonal antibody, were carried out with genomic DNA from CTRL-C1 and AOA2-P1 fibroblasts. R-loop prone regions at *WWOX* and *CDH13* genes were analyzed. Relative values of R-loops immunoprecipitated in each region, normalized to input values and to the signal at the *SNRPN*-negative control region, are shown. Data represent the mean ± SEM of three independent experiments. (*G*) ChIP-qPCR analyses at *WWOX* and *CDH13* genes using a γ-H2AX antibody was carried out with cross-linked chromatin from CTRL-C1 and AOA2-P1 fibroblasts. Fold-enrichment was calculated as a ratio of γ-H2AX antibody signal versus control IgG. Data represent the mean ± SEM of three independent experiments.

Copy number changes (CNCs), such as submicroscopic deletions (losses) and amplifications (gains), can be caused by transcription-dependent genomic instability, as identified using array comparative genomic hybridization (aCGH) (33, 34). To determine whether SETX-deficient cells exhibit CNCs, genomic DNAs extracted from AOA2-P1 and CTRL-C1 cells were compared by aCGH using whole-genome arrays containing 400K unique oligonucleotide sequences. We observed 57 and 48 CNCs in two independent experiments (Fig. 2*A* and *SI Appendix*, Fig. S2*A*), and of these 47 CNCs (23 gains and 24 losses) were common to both experiments (Fig. 2*B*). Remarkably, 63% of the CNCs were located in regions containing known genes, indicating their association with transcription (Dataset S1). Permutation-based overlap comparisons of CNCs and previously identified fragile sites revealed that the gains, but not losses, were enriched for early replicating fragile sites (ERFS) (35), which colocalize with transcriptionally active gene clusters with high GC content (Fig. 2*C* and *SI Appendix*, Fig. S2*B*). Notably, 15 gains and 11 losses overlapped with common/rare fragile sites (C/RFS) (36), and many of the genes in these regions mapped to common fragile sites (CFSs) (37), such as *CDH13*, *PARK2*, and *WWOX* (Dataset S1). We were, however, unable to confirm the statistical significance of this overlap with permutation-based tests, likely because CFSs and C/RFSs cover large portions of the genome (*SI Appendix*, Fig. S2 *C–E*).

SETX was recently shown to be recruited to DSBs when they occur in transcriptionally active loci and resolve R-loops formed at DSBs to prevent translocations (29). Consistent with this, gain regions were significantly enriched for genomic locations of DSBs induced by neocarzinostatin (NCS-breakome) (36) (Fig. 2*D* and *SI Appendix*, Fig. S2*B*), but not those induced by aphidicolin (APH-breakome) that were nonuniformly distributed along the genome (38) (Fig. 2*E* and *SI Appendix*, Fig. S2*B*).

CFSs represent unusually long genes that are difficult to transcribe and replicate, where collisions between the replisome and transcription apparatus lead to R-loop formation and DNA breakage. We therefore used DNA–RNA immunoprecipitation (DRIP) to investigate the accumulation of R-loops at two CFS loci (*WWOX* and *CDH13*) in genomic DNA from the AOA2-P1 and CTRL-C1 fibroblasts. Quantitative real-time PCR (qPCR) analyses revealed increased R-loop formation at both loci in AOA2-P1 compared to the CTRL-C1 (Fig. 2*F*). Moreover, these CFS loci accumulated significant levels of DNA damage, as measured by γ-H2AX chromatin immunoprecipitation (ChIP) (Fig. 2*G*).

**Fig. 3.**
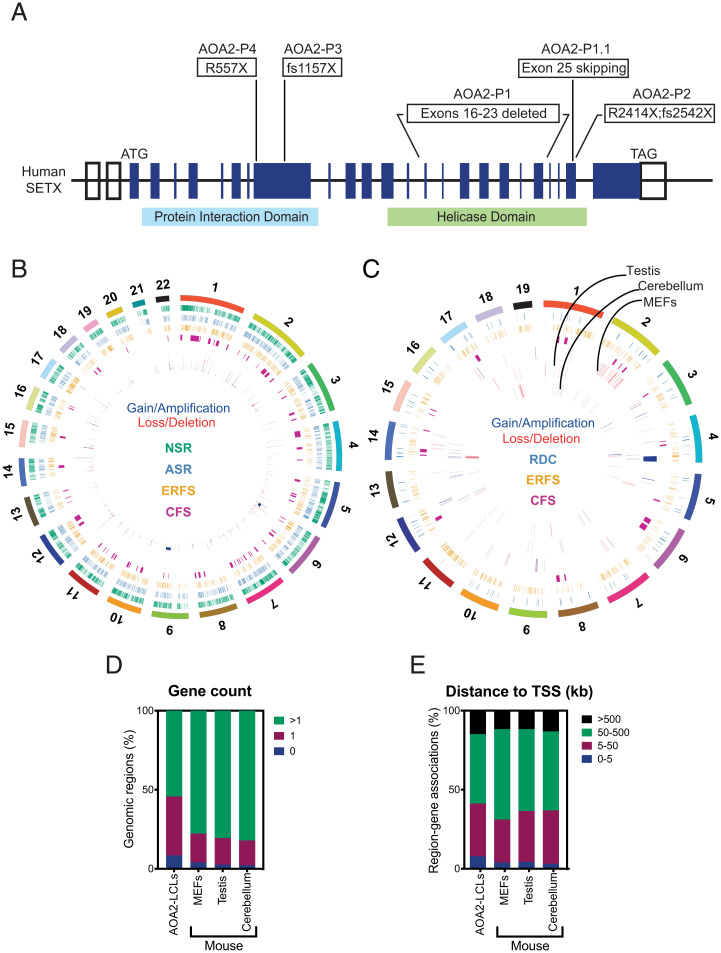
Hotspots of genomic instability in SETX-deficient human and mouse cells. (*A*) Diagram of human *SETX* showing the mutations in the AOA2 LCLs. (*B*) Circos plot depicting the identified CNCs in AOA2 LCLs. Gains/amplifications and losses/deletions are marked in blue and red, respectively. Chromosome locations of known fragile sites are marked in magenta (CFS), orange (ERFS), cyan (ASR), and green (NSR). The outer ring shows all chromosomes. (*C*) As in *B*, except that the Circos plot depicts CNCs identified in MEFs, testis, and cerebellum of *Setx*^−/−^ mice. RDC, recurrent DNA break cluster. (*D*) Genomic Regions Enrichment of Annotations Tool (GREAT) analyses were carried out with aCGH datasets from AOA2-LCLs, mouse cells, and tissues (MEFs, testis, and cerebellum). Plot depicts the percentage of genomic regions associated with the indicated gene count. (*E*) Plot depicting the distance of genomic regions to their nearest TSSs, identified in AOA2 and mouse aCGH experiments. Distances are indicated in kilobases.

Τo confirm that loss of SETX induces genome-wide CNCs, aCGH was used to compare genomic DNA from five different patient-derived AOA2 lymphoblastoid cell lines (LCLs; P1 to P4 and P1.1) (Fig. 3*A*) with their respective controls (C1 to C4 and C1.1). A significant number of CNCs (369 gains and losses) were identified (Fig. 3*B*, *SI Appendix*, Fig. S3*A*, and Dataset S2) and mapped mainly to gene-rich regions (81%) (Fig. 3 *B*, *D*, and *E*). Overlap analyses revealed that both gain and loss regions were significantly enriched for GC-rich and transcriptionally active ERFS (*P* = 8 × 10^−06^ and *P* = 1.9 × 10^−02^, respectively, by permutation test), and only loss regions were significantly enriched for NCS-breakome sensitive regions of the genome (*P* = 4.3 × 10^−03^ by permutation test) (Fig. 3*B*). Overlap tests revealed no significant enrichment of the APH-breakome sensitive regions (ASR). Consistent with our findings in AOA2-P1 fibroblasts, we identified 78 gains and 81 losses that overlapped with the C/RFSs, and 34 gains and 37 losses that overlapped with CFSs.

To confirm and extend these results, aCGH experiments were performed using genomic DNA isolated from mouse embryonic fibroblasts (MEFs), testis, and cerebellar vermis isolated from *Setx^+/+^ and Setx^−/−^* KO mice (28 d old) and the results compared with the list of genomic gains and losses (Fig. 3*C*, *SI Appendix*, Fig. S3 *B–D*, and Dataset S3). Consistent with the AOA2 patient-derived cell data, the CNCs mapped to gene-rich regions (Fig. 3 *D* and *E*). Strikingly, both in MEFs and cerebellum, the gain regions were significantly enriched for Ensembl genes (*P* = 6.5 × 10^−11^ and *P* = 1.3 × 10^−02^, respectively, by permutation test). In MEFs, but not in testis and cerebellum, the gain regions were significantly enriched for ERFS (*P* = 2.6 × 10^−11^), which is in agreement with the AOA2-P1 fibroblast data. In cerebellum, 27% of the loss regions were associated with CFSs, whereas none of the gains/losses from MEFs overlapped with CFSs (Fig. 3*C*). In addition, some of the mouse cerebellum CNCs overlapped with recurrent DNA break clusters identified in long neural genes from neural stem/progenitor cells (Fig. 3*C*) (39, 40).

Collectively, these results show that loss or inactivation of SETX gives rise to genome-wide chromosomal fragility associated with the transcribed regions of the mammalian genome.

### Differential Gene Expression in SETX-Deficient Human and Mouse Cells.

Because CNCs can affect the cellular transcriptome (34), gene-expression profiling of the AOA2 (P1 to P4) and CTRL (C1 to C4) LCLs was carried out by microarray analysis. A significant number of genes (ranging from 365 to 675 genes) showed a greater than twofold change (*P* < 0.05) in expression in the AOA2 LCLs. Comparison of AOA2 LCLs with their controls revealed a total of 832 up-regulated and 656 down-regulated genes in AOA2 (Dataset S4). Strikingly, the expression of 310 genes (209 and 101 genes were up-regulated and down-regulated, respectively) was significantly altered in all four AOA2 cell lines. Among these, 27 up-regulated and 12 down-regulated genes correlated with genomic regions that scored as gains and losses, respectively.

To identify the genes most affected by *SETX* mutations, we carried out RNA sequencing (RNA-seq) with AOA2 (P1 to P4) and CTRL (C1 to C4) LCLs and found numerous genes that were either up or down-regulated (*SI Appendix*, Fig. S4 *A–D* and Dataset S5). Comparison of the AOA2 LCLs revealed a core set of 96 genes (55 up-regulated and 41 down-regulated; greater than twofold change) that exhibited the greatest expression differences relative to the controls (*SI Appendix*, Fig. S4*E*). Furthermore, gene set enrichment analysis, using the ranked test statistics from the differential expression analysis of CTRL and AOA2 LCLs, revealed that transcription from RNAPII promoters in response to stress, RNA splicing, regulation of RNA stability and RNA 3′-end processing were significantly (false-discovery rate < 0.05) enriched (Dataset S6).

Differential gene-expression profiles were also confirmed with *Setx^−/−^* KO mouse MEFs (684 up-regulated genes and 470 down-regulated genes) by RNA-seq (*SI Appendix*, Fig. S4*F* and Dataset S5). Importantly, overlap analyses revealed that the gain regions identified in *Setx^−/−^* MEFs were enriched for up-regulated genes (*P* = 1.2 × 10^−08^ by permutation test), while loss regions did not show significant overlap with down-regulated genes. Taken together, these data indicate that CNCs can contribute to the gene-expression changes identified in the absence of SETX.

### Loss of SETX Alters the Profile of RNAPII Across the Genome.

**Fig. 4.**
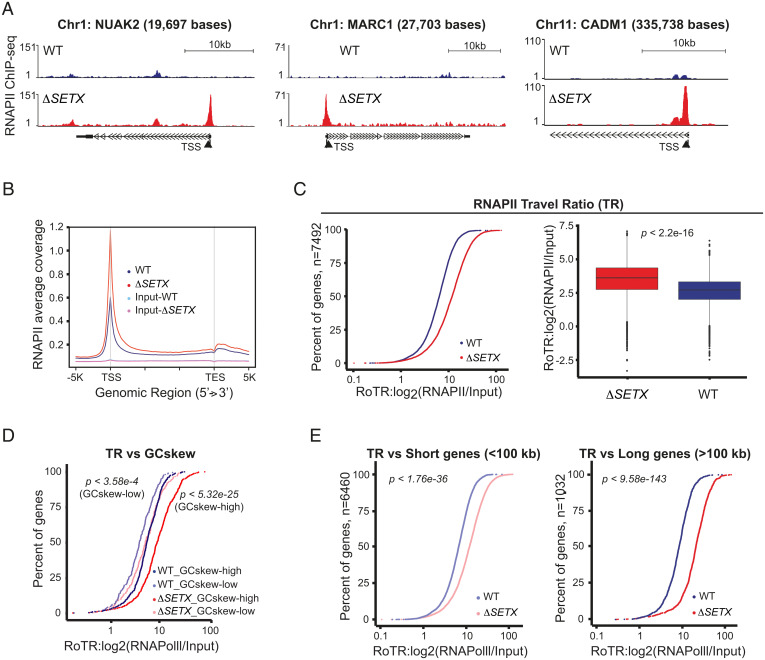
Loss of SETX causes transcription stress. (*A*) ChIP-seq analyses using RNAPII antibodies were carried out with cross-linked chromatin from HAP1 WT and Δ*SETX* cell lines. Representative genome browser shots of RNAPII binding over the *NUAK2*, *MARC1*, and *CADM1* genes are shown. (Scale bars, 10 kb.) (*B*) Average RNAPII ChIP-seq coverage profiles for WT and Δ*SETX* are shown, along with input controls. TES, transcription end site. (*C*, *Left*) Cumulative curve of RNAPII ratio of traveling ratios (RoTR; *x* axis) for 7,492 genes in WT and Δ*SETX*. The *y* axis indicates percent of all genes. (*Right*) Box plots show the distribution of RoTR of 7,492 genes in WT and Δ*SETX*. *P* values were calculated using nonparametric Wilcoxon rank sum test. (*D*) Cumulative curve of RoTR in WT and Δ*SETX* with GCskew-high (*n* = 505) and GCskew-low (*n* = 215) groups that were called based on a 10% and 90% quantile from the GCskew frequency distribution of the entire genic intervals (*n* = 26,934). *P* values were calculated as in *C*. (*E*, *Left*) Cumulative curve of RoTR of 6,460 short genes (<100 kb) in WT and Δ*SETX.* (*Right*) Cumulative curve of RoTR of 1,032 long genes (>100 kb) in WT and Δ*SETX*. *P* values were calculated as in *C*.

To investigate the role of SETX in transcription, RNAPII ChIP-sequencing (ChIP-seq) was carried out with human HAP1 WT and *SETX* KO (Δ*SETX*) cells. We observed a genome-wide increase in RNAPII levels over transcription start sites (TSS) in the Δ*SETX* cells (Fig. 4 *A* and *B*). To quantitatively analyze how SETX affects transcription at the genomic level, we measured the traveling ratio (TR) that compares the ratio between RNAPII density in the promoter-proximal region (−30 to +300 bp relative to the TSS) and the remaining length of the gene (41). The TR was found to be globally higher in Δ*SETX* cells compared to WT and input controls (*SI Appendix*, Fig. S5 *A* and *B*). When the analysis was focused on the 7,492 genes (Dataset S7) with clear RNAPII peaks at the promoter-proximal region (active genes), we observed that these genes exhibited a higher TR in Δ*SETX* compared to WT (Fig. 4*C* and *SI Appendix*, Fig. S5*C*). Together, these data show that SETX inactivation affects RNAPII progression throughout the genome, and most likely causes frequent RNAPII pausing/stalling (transcription stress) over the TSS.

Base composition analyses revealed significantly higher GC-richness and GCskew at the promoter-proximal regions of these 7,492 genes (*SI Appendix*, Fig. S5*D*). To investigate how GCskew affects TR, we calculated GCskew of genic regions (*n* = 26,934), and subsequently “GCskew-high” and “GCskew-low” groups were called based on a 10% and 90% quantile from the distribution of the GCskew values. Strikingly, the differences in TR between Δ*SETX* and WT was significantly higher for GCskew-high genes (*n* = 505) compared to GCskew-low (*n* = 215) genes (Fig. 4*D*).

Previously, it was shown that transcription through regions of GCskew leads to the formation of long and stable R-loops (2). Consistent with this, DRIP analyses revealed the accumulation of R-loops at six different genes (*NUAK2*, *CADM1*, *TRIM33*, *SOD1*, *MARC1*, and *DDIT4L*) identified to have transcription stress in Δ*SETX* (*SI Appendix*, Fig. S5*E*). Importantly, RNaseH treatment, but not RNaseA treatment, abolished R-loop accumulation. The negative control gene, *SNRPN*, did not exhibit R-loop accumulation.

Because long genes (CFSs) and highly transcribed short genes (e.g., histone genes) are prone to transcription stress and R-loop formation, we next focused on the relationship between gene length and TR of the 7,492 genes. We classified 6,460 and 1,032 genes as short (<100 kb) and long (>100 kb) genes, respectively. We found that the TR was significantly higher for both long and short genes in Δ*SETX* compared to WT (Fig. 4*E*), although the TR difference was greater in long compared with short genes. Similar results were observed when the genes were stratified into short (shortest 20%), medium (middle 40 to 60%), and long (longest 20%) groupings based on quantiles from the gene-width distributions. The results confirmed that the TR differences were higher in long genes (*SI Appendix*, Fig. S5*F*). In agreement with the findings from AOA2 and *Setx^−/−^* MEFs, differential gene-expression profiles were also observed in human HAP1 Δ*SETX* cells (248 up-regulated genes and 364 down-regulated genes) by RNA-seq analyses (*SI Appendix*, Fig. S4*G* and Dataset S5).

Collectively, these data lead us to conclude that SETX controls the movement of RNAPII across genes. In the absence of SETX, cells exhibit elevated levels of transcription stress and increased R-loop accumulation, particularly near high GCskew promoter-proximal regions which affects gene expression.

### Transcription Stress Correlates with Chromosome Instability upon SETX Deficiency.

**Fig. 5.**
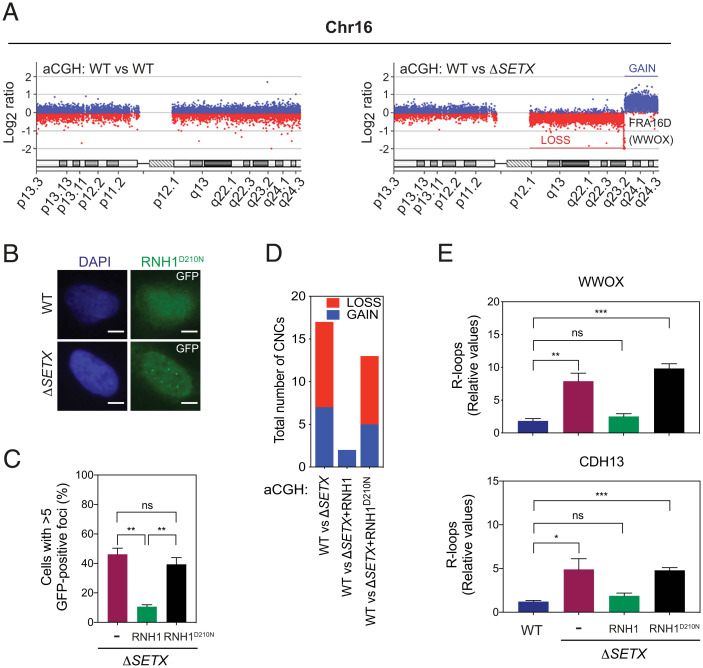
SETX suppresses transcription stress that promotes chromosome instability. (*A*) aCGH profile of chromosome 16 (Chr16) in WT versus WT condition (*Left*) and WT versus Δ*SETX* (*Right*). The WWOX/FRA16D locus at q23.1 is lost. Other gain and loss regions are marked in blue and red lines, respectively. (*B*) Detection of R-loops in WT and Δ*SETX* cells by immunofluorescence using catalytically inactive RNaseH1 (RNH1^D210N^) fused to GFP. Nuclear DNA was stained with DAPI (blue). Representative images are shown. GFP, green fluorescent protein. (Scale bars, 5 μm.) (*C*) Quantification of GFP^+^ RNH1 foci in Δ*SETX* cells transfected with RNH1 or RNH1^D210N^. Data represent the mean ± SEM of three independent experiments with >100 cells per condition. **P < 0.01. (*D*) aCGH analyses were carried out with WT vs. Δ*SETX*, WT vs. Δ*SETX* + RNH1 and WT vs. Δ*SETX* + RNH1^D210N^. The graph depicts the number of CNCs identified in each experiment. Chromosome regions with gain (blue) and loss (red), are indicated. Data represent the mean of two independent experiments. (*E*) DRIP-qPCR analysis using genomic DNA from WT, Δ*SETX*, Δ*SETX* + RNH1 and Δ*SETX* + RNH1^D210N^. R-loop regions at *WWOX* (*Upper*) and *CDH13* (*Lower*) were analyzed. Data represent the mean ± SEM of three independent experiments.

To confirm and extend the results observed with AOA2 LCLs, we measured CNCs in the Δ*SETX* cell line and identified 17 and 14 genomic abnormalities of which 13 (9 losses and 4 gains) were common to the two independent experiments (*SI Appendix*, Fig. S6*A* and Dataset S8). Consistent with them arising from chromosome instability, 11 of the 13 CNCs mapped to genes and 6 coincided with CFSs. Genomic abnormalities observed in Chromosome 16 in Δ*SETX* encompassing the *FRA16D* locus (*WWOX*) is shown in Fig. 5*A*. Others were generally located in gene-rich regions and showed increased RNAPII pausing at gene promoters. The genomic gain at chromosome 17 is shown as an example (*SI Appendix*, Fig. S6*B*).

SETX-related genomic instability was associated with the formation of R-loops as determined by immunofluorescent analysis in Δ*SETX* cells overexpressing a catalytic-inactive mutant of GFP-tagged RNaseH1 (RNH1^D210N^) (Fig. 5 *B* and *C*). Consistent with this, 13 genomic alterations (8 losses and 5 gains) were identified in two independent aCGH experiments carried out with WT vs. Δ*SETX* + RNH1^D210N^ cells (*SI Appendix*, Fig. S6*A*). Eleven of these were similar to aberrations identified in the earlier aCGH experiments with WT vs. Δ*SETX* (Fig. 5*D*). In contrast, aCGH experiments with WT vs. Δ*SETX* + RNH1 revealed only two nonspecific (both in Y chromosome) genomic aberrations. DRIP analysis of the *WWOX* and *CDH13* fragile site loci confirmed the accumulation of R-loops in Δ*SETX and* Δ*SETX*+RNH1^D210N^ cells, but not in Δ*SETX* + RNH1 cells (Fig. 5*E*). Furthermore, these CFS loci accumulated significant levels of DNA damage, as measured by γ-H2AX ChIP analysis (*SI Appendix*, Fig. S6*C*). Together, these results confirm that transcription stress causes chromosome instability in cells lacking SETX.

### TCR Promotes Genomic Instability.

**Fig. 6.**
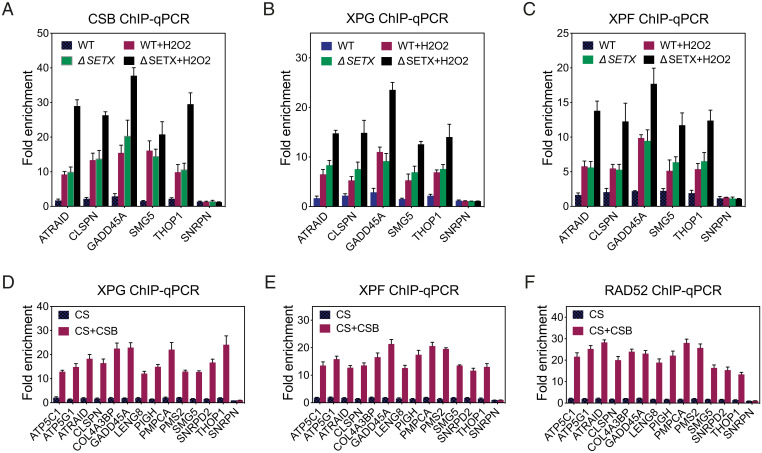
CSB promotes aberrant repair of TSS-associated R-loops in the absence of SETX. (*A*) ChIP assays were carried out using cross-linked chromatin from HAP1 WT and Δ*SETX* cells treated with or without H_2_O_2_. Rabbit polyclonal antibody against CSB was used for ChIP. TSS regions from the indicated genes were analyzed by qPCR. Data represent the mean ± SEM of three independent experiments. (*B*) As in *A*, using XPG monoclonal antibody. (*C*) As in *A*, using XPF rabbit polyclonal antibody. (*D*) As in *B*, except cross-linked chromatin from CS1ANsv (CS) and CS cells stably expressing GFP-tagged CSB from a BAC (CS + CSB) were used for ChIP. (*E*) As in *D*, using XPF rabbit polyclonal antibody. (*F*) As in *D*, using RAD52 rabbit polyclonal antibody.

Unscheduled R-loops that form cotranscriptionally are prone to DNA breakage and genomic rearrangements, in reactions that involve TCR proteins (12, 42, 43). We therefore investigated whether TCR proteins were recruited to the TSS of RNAPII-transcribed genes that showed elevated RNAPII pausing and increased R-loop formation upon SETX deficiency. In five genes tested, ChIP analysis revealed an enrichment of the TCR proteins CSB, XPG, and XPF at the TSS in Δ*SETX* compared to WT cells (Fig. 6 *A–C*). Treatment of the cells with H_2_O_2_ further increased the occupancy of TCR proteins at these sites (Fig. 6 *A–C*), consistent with the observation that oxidative stress increases the accumulation of promoter paused RNAPII (44). Using Cockayne syndrome (CS) and CSB-complemented cells (CS+CSB), we found that the absence of CSB resulted in a failure to recruit XPG and XPF endonucleases for the processing of R-loops at the TSS of genes containing paused RNAPII (Fig. 6 *D* and *E*). In addition, ChIP analyses revealed that the absence of CSB led to a failure to recruit RAD52 protein to these TSSs (Fig. 6*F*), consistent with RAD52’s involvement in the recombinational repair of actively transcribed regions by transcription-coupled homologous recombination (45, 46).

**Fig. 7.**
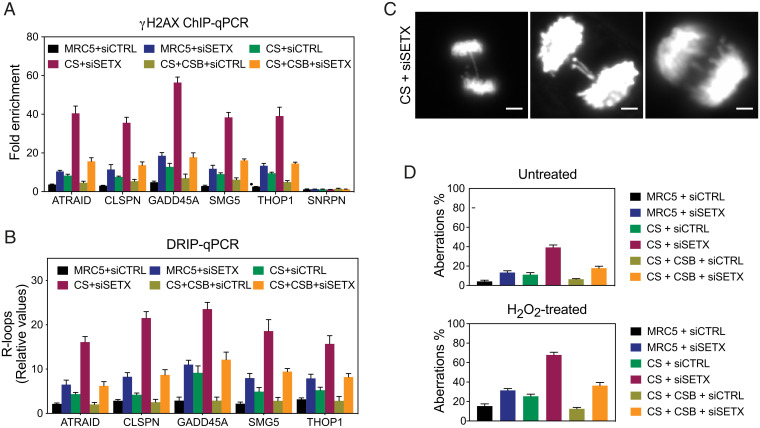
CSB induced DNA damage in SETX-deficient cells. (*A*) ChIP assays were carried out using cross-linked chromatin from the indicated cells treated with control siRNA (siCTRL) or *SETX* siRNA (siSETX). TSS regions from the indicated genes were analyzed by qPCR. Data represent the mean ± SEM of three independent experiments. (*B*) DRIP assays were carried out using cross-linked chromatin from the indicated cells treated with siCTRL or siSETX. TSS regions from the indicated genes were analyzed by qPCR. Data represent the mean ± SEM of three independent experiments. (*C*) Representative images of chromosome aberrations (bulky DNA bridges and lagging chromosomes) in SETX-depleted CS cells. DNA was stained with DAPI. (Scale bars, 5 μm.) (*D*) Quantification of the percentage of chromosome aberrations in the indicated cells treated with or without H_2_O_2_. Three-hundred mitotic cells were analyzed. Data represent the mean ± SEM of three independent experiments.

To further understand the relationships between TCR and SETX in R-loop processing, we determined the levels of DNA damage at the TSS regions of genes with paused RNAPII. ChIP analyses revealed an enrichment of γH2AX at the TSS of these genes in SETX-depleted MRC5 cells compared to controls (Fig. 7*A*). We also detected increased levels of γH2AX at the TSS of these genes in CS, but not CS complemented (CS+CSB) cells. The levels of γH2AX were consistently reduced in CS cells compared with SETX-depleted cells, and depletion of SETX from the CS cells led to a further increase in γH2AX (Fig. 7*A*). Importantly, the levels of γH2AX accumulation detected in control or SETX-depleted MRC5, CS, and CS+CSB cells correlated with the levels of R-loops at the TSS of these genes (Fig. 7*B*). These results indicate that the TCR pathway and SETX act independently to remove R-loops at TSS of RNAPII-transcribed genes. Consistent with this, SETX-depleted CS cells exhibit elevated levels of DNA anaphase bridges and lagging chromosomes (Fig. 7 *C* and *D*). The occurrence of these chromosome aberrations was further increased upon H_2_O_2_ treatment that stabilizes promoter-paused RNAPII (Fig. 7*D*).

## Discussion

Our results demonstrate that the loss of SETX causes transcription stress, increased R-loop formation, and chromosomal instability at RNAPII-transcribed genes and fragile sites. SETX promotes R-loop repair to remove R-loops that form near the promoters of RNAPII-transcribed genes, and thereby controls transcription and maintains genome stability. In the absence of SETX, aberrant R-loops are incised by TCR nucleases leading to DNA damage and genomic instability (*SI Appendix*, Fig. S7). Consistent with previous work (12), we observed that loss of SETX leads to DNA breaks that result in genomic aberrations, such as amplifications/gains or deletions/losses. We found that gain regions colocalized with a large proportion of the ERFS, which were found within the transcriptionally active gene clusters with high GC content that replicate early. In contrast, losses were largely associated with long genes/CFSs, where transcription happens throughout the cell cycle.

Active gene promoters represent major hotspots for R-loop formation, as ∼60% of R-loops map to promoter-proximal regions (2, 4, 47, 48). We have shown that SETX acts at the promoter-proximal regions of RNAPII-transcribed genes to control transcription stress-induced R-loop accumulation and prevent genome-wide chromosomal instability. Our results are consistent with observations showing that promoters containing R-loops, which arise due to SETX-deficiency are resistant to cytosine methylation by DNA methyltransferase 1 (DNMT1) and exhibit transcriptional dysregulation (22). The underlying basis for this lack of methylation is that DNMT1 preferentially binds double-stranded DNA, but not RNA–DNA hybrids (22). SETX-mediated R-loop removal is particularly important for chromosome stability at CFSs, long genes, and highly transcribed genes, all of which are prone to replication/transcription stress. The mis-regulation of vital transcripts, in addition to the observed chromosome instability, may contribute to disease progression in AOA2.

The fate of paused RNAPII remains enigmatic. Early observations led to the proposal that RNAPII backtracking at pause sites provides a free RNA 3′ end for the RNA exosome, resulting in degradation of the RNA transcript and transcription termination (49). However, increased RNAPII pausing and backtracking also leads to R-loop formation and genome instability (50, 51). Transcription problems arising due to backtracking are resolved by TFIIS (also known as TCEA1) and intrinsic transcript cleavage by RNAPII. Using TFIIS mutant cells, it was shown that R-loops form at the anterior side of backtracked RNAPII and trigger genome instability (51). Possible interactions between TFIIS and SETX could provide a link between RNAPII backtracking and the R-loop resolution machinery, supporting the notion that SETX may play an important role in the removal of R-loops formed at the anterior side of RNAPII.

In yeast and mammals, Sen1/SETX facilitates pause-dependent transcriptional termination at specific RNAPII-transcribed genes (9, 10, 52–54). Transcription termination requires a functional polyadenylation signal (PAS) and either downstream pause sites or cotranscriptional cleavage sequences together with 3′ transcript degradation by the 5′-3′ exonuclease Rat1 (in budding yeast) and XRN2 (in humans) (55–59). Interestingly, the terminator regions of PAS-dependent genes, which are devoid of GC skewness but contain G-rich pause sites, facilitate RNAPII pausing and contribute to R-loop formation (4). These R-loop structures are resolved by SETX and the nascent RNA is degraded by XRN2 leading to efficient transcription termination (10).

Genome instability in the adult brain leads to impaired neural development and neurodegeneration (60, 61). The central nervous system is particularly vulnerable to oxidative stress due to the high levels of oxygen consumption, low levels of antioxidant enzymes and the terminally differentiated state of neurons. Indeed, oxidative stress represents a major cause of neuropathology underlying a variety of neurodegenerative diseases. Moreover, DNA breaks are associated with cognitive impairment in neurodegenerative conditions, such as Alzheimer’s disease and ALS. Consequently, mutations in many DNA single- and double-strand break repair proteins have been linked to multiple neurodegenerative diseases, including other subtypes of AOA. Recent studies indicate that oxidative stress (i.e., treatment with a low dose of H_2_O_2_) causes a rapid and genome-wide increase (up to fivefold) in promoter-proximal paused RNAPII (44). These observations are consistent with the results presented here and provide an explanation for the hypersensitivity of AOA2 cells to H_2_O_2_ treatment, together with altered gene-expression profiles and genomic instability.

In conclusion, our findings indicate that AOA2 is a transcription stress-related disorder and that SETX is necessary to preserve the integrity of transcription. The absence of SETX leads to R-loop accumulation and aberrant resolution by the TCR endonucleases XPG and XPF, and RAD52 recombination protein, leading to DNA damage and genomic instability. Targeting of these proteins is dependent upon CSB. Other neurological diseases, including tremor-ataxia syndrome, autosomal dominant proximal spinal muscular atrophy, and Charcot-Marie-Tooth disease have also been linked with mutations in the *SETX* gene (62–64). Further studies will provide invaluable insights that will allow us to piece together the relationships between transcription stress and human disease.

## Methods

### Cell Culture.

All cells and materials are listed in *SI Appendix*, Table S1. MRC5 (MRC5VA), CS (CS1AN_SV_; CSB fibroblast SV40 immortalized), and CS + CSB (CS cells stably expressing GFP tagged CSB from a BAC) cells were cultured in DMEM (ThermoFisher) supplemented with 10% fetal bovine serum (FBS) and 1% penicillin/streptomycin (P/S). U2OS cells stably expressing GFP tagged mouse 53BP1 (U2OS-GFP-m53BP1), were grown in DMEM supplemented with 10% FBS, 1% P/S, and 400 μg/mL G418 (ThermoFisher). Human HAP1 WT and Δ*SETX* cells were grown in IMDM medium (ThermoFisher) plus 10% FBS and 1% P/SLCLs from control and AOA2 patients were cultured in RPMI medium 1640 (ThermoFisher) containing 20% FBS, 1% P/S, and 2 mM l-glutamine (ThermoFisher). Human foreskin fibroblasts (HFF; control) and fibroblasts from an AOA2 patient were grown in DMEM supplemented with 15% FBS and 1% P/S. All human cell lines were grown at 37 °C and in a humidified atmosphere containing 5% CO_2_. *Setx*^+/+^ and *Setx*^−/−^ MEFs were cultured at 37 °C with 5% CO_2_ and 5% O_2_ in DMEM supplemented with 15% FBS (Sigma) and 1% P/S. Murine tissues were prepared as previously described (21). All animal experiments were approved by the QIMR Berghofer Medical Research Institute Animal Ethics Committee, The University of Queensland, Australia.

### MEF Isolation and Culture.

For generation of *Setx*^−/−^ MEFs, *Setx*^+/−^ parent mice were combined for 12 h for copulation. Pregnant females at 13.5 d gestation were subjected to killing under anesthesia. Individual embryos were collected by uterine dissection, rinsed in PBS followed by removal of head, heart and liver. The remaining embryo was minced using sterile scalpel blades and incubated in trypsin at 37 °C for 20 min. The tryptic digest was centrifuged and the supernatant discarded. Pelleted cells were suspended in DMEM, 15% FBS, and 1% P/S and plated for cell culture. Cells were maintained using a standard 3T3 protocol.

### Plasmids.

A short-guide RNA (sgRNA) targeting a coding sequence of exon 4 in *SETX* was inserted into the BbsI site of pX330 (plasmid 42230, Addgene) as described previously (65, 66). The sgRNA oligonucleotides are listed in *SI Appendix*, Table S1. The plasmid EGFP-N2 containing hM27RNaseH1 (GFP-RNH1) (67), lacking a mitochondrial localization sequence, was provided by Robert J. Crouch (NIH, Bethesda, MD). The catalytic dead mutant of RNH1, RNH1^D210N^, was generated using the Quikchange Site-Directed Mutagenesis Kit (Agilent).

### Transfection.

Transfection of small-interfering RNA (siRNA) was carried out using Lipofectamine RNAiMAX according to the manufacturer’s instructions (Invitrogen). For siRNA transfection, cells were seeded at 30% confluence, grown for 24 h, and transfected at a final siRNA concentration of 40 nM. Transfection was repeated 24 h after the first transfection. Unless indicated otherwise, cells were analyzed 72 h after the first transfection. For plasmid transfection, cells were seeded at 50% confluence, incubated for 24 h, and transfected with 2 to 4 μg of plasmid DNA using Lipofectamine 2000. Unless indicated otherwise, cells were analyzed 48 h after transfection.

### Cell Line Manipulation and Generation.

To generate *SETX* KOs in the HAP1 line, cells were transiently transfected with pX330 containing a *SETX*-specific sgRNA sequence, along with pSUPER.puro (Oligoengine) at a 7:1 ratio. After 24 h, cells were selected with 1 μg/mL puromycin (ThermoFisher) for 2 d before the cells were harvested and seeded as single colonies. Initially, clones were selected on the basis of a negative signal for SETX by Western blotting. The selected clones were then sequence-verified.

### Western Blotting.

Whole-cell lysates were subjected to SDS/PAGE and analyzed by Western botting. The following primary antibodies were used: rabbit anti-SETX (ABN421; 1:2,000), rabbit anti-CSB (A301-345A; 1:1,000), rabbit anti-GFP (ab290; 1:2,000), and mouse anti–β-Tubulin (sc-5274; 1:2,000). The secondary antibodies used were goat anti-rabbit immunoglobulins/HRP and goat anti-mouse immunoglobulins/HRP (Dako).

### Immunofluorescence.

Cells grown on glass coverslips were fixed with PTEMF buffer (20 mM Pipes pH 6.8, 0.2% Triton X-100, 10 mM EGTA, 1 mM MgCl_2_ and 4% formaldehyde [methanol-free]) for 10 min at room temperature. Fixed cells were then washed with PBS, blocked in 5% BSA/PBS for 45 min followed by incubation with primary antibody in 5% BSA/PBS overnight at 4 °C. The next day, the coverslips were washed with PBS and incubated with the secondary antibody for 1 h at room temperature. After washing with PBS, coverslips were mounted with Prolong Gold Antifade Mounting medium containing DAPI (ThermoFisher) and the coverslip edges were sealed with nail polish. After air-drying, images were acquired using Volocity software on an Axio Imager M2 microscope (Zeiss) equipped with an ORCA-ER camera (Hamamatsu) and a Plan-SPOCHROMAT 63×/1.4 oil objective. Images were processed using ImageJ software. For double-immunostaining experiments (53BP1/Cyclin A) at least 100 nuclei were scored in each experiment. For the analysis of DAPI-positive chromosome bridges and lagging chromosomes, at least 50 anaphase cells were scored in each experiment. The primary antibodies used were rabbit anti-53BP1 (ab36823; 1:500) and mouse anti-Cyclin A (sc-56299; 1:200). Secondary antibodies conjugated to Alexa Fluor 488 and Alexa Fluor 568 were used for immunodetection.

### Micronucleus Formation.

Cells grown on glass coverslips were treated with or without cordycepin. For quantification of micronuclei, cells were then treated with cytochalasin B (Sigma-Aldrich; 2 μg/mL for 16 h) to block cells in cytokinesis and fixed with PTEMF buffer for 10 min at room temperature. Slide mounting and imaging acquisition were performed as described above. At least 100 DAPI-stained binucleated cells were scored for the presence or absence of micronuclei in each experiment.

### Live-Cell Imaging.

U2OS-GFP-m53BP1 cells (a gift from Jiri Lukas, University of Copenhagen, Denmark) were seeded in Labtek chambers (Nunc, ThermoFisher) and after 24 h were transfected with either a *SETX*-targeting siRNA or a nontargeting negative control siRNA. After 36 h of siRNA transfection, the culture medium was changed to CO_2_-independent medium without phenol red (ThermoFisher) and live-cell imaging was carried out as described previously (27).

### Imagestream Analysis.

Cells were trypsinized and fixed in 70% ethanol for 1 h at 4 °C. They were then washed and resuspended at 2 × 10^7^ cells/mL in PBS containing 2% FBS. Cells were supplemented with 1 μg/mL propidium iodide (PI) and labeled for 15 min at room temperature. Flow cytometry and data collection was performed using the ImageStream^X^ Mark II Imaging Flow Cytometer and Inspire acquisition software. Cells were imaged using channel 1 for bright field and fluorescent channel 5 (Bands 640 to 745 nm) and excitation laser 561 at 150 to 200 mW. Image analysis was performed using the IDEAS software v6.2. Single cells were filtered using a scatter plot of bright field aspect ratio and cell area. In-focus cells were filtered using a root mean square (RMS) histogram with Gradient RMS > 50 within the bright field parameter. PI signal was inspected using the intensity channel 5 M05. Cells were inspected manually for micronuclei formation.

### M-FISH.

Human 24-color M-FISH were performed as described previously (68). At least 30 metaphases from each sample were karyotyped based on M-FISH classification and DAPI-banding pattern.

### ChIP.

ChIP experiments were performed using the ChIP-IT Express Kit (Active Motif) as described previously (69). ChIP-derived DNA was purified using either the QIAquick PCR Purification Kit (Qiagen) or the Chromatin IP DNA Purification Kit (Active Motif). The following primary antibodies were used: rabbit anti-γH2AX (phospho S139; ab2893), rabbit anti-RPA (ab10359), rabbit anti-53BP1 (NB100-304), rabbit anti-CSB (A301-345A), rabbit anti-XPF (sc-28718), mouse anti-XPG (8H7) and mouse anti-RAD52 (sc-365341).

### DRIP.

DRIP experiments were performed essentially as described previously (2).

### qPCR.

ChIP- and DRIP-derived DNA samples were subjected in triplicates (1.5 to 2 µL) to qPCR analysis on a CFX96 Real-Time Analyzer (Bio-Rad) using iQ SYBR Green Supermix reagent (Bio-Rad) and the gene-specific primers are listed in Dataset S7. The data were analyzed using the 2^−ΔΔCT^ method (70). Immunoprecipitated DNA was calculated as the percentage of DNA in the immunoprecipitates compared with input DNA. Fold-enrichment of each target region was calculated as the ratio of the amounts of immunoprecipitated DNA estimated for the desired antibody versus control IgG.

### Gene-Expression Arrays.

Total RNA was purified using RNeasy Kit (Qiagen) from AOA2-mutant and respective control cells, according to the manufacturer’s instructions. Each condition was represented by three biological replicates. RNA samples were processed at the CRUK Manchester Institute using the NuGEN Amplification Kit (NuGEN) followed by hybridization to Affymetrix Human Gene 1.0 ST Array for gene expression. Arrays were scanned using the Affymetrix GeneArray 3000 7G scanner.

### Array Comparative Genomic Hybridization.

Genomic DNA from cultured human cells were isolated using the QIAamp DNA kit (Qiagen) and genomic DNA from MEFs and mouse tissue samples were extracted using the DNeasy Blood and Tissue Kit (Qiagen), according to the manufacturer’s recommendation. The Agilent SurePrint G3 Human CGH Microarrays 2 × 400K and the Agilent SurePrint G3 Mouse CGH Microarrays 1 × 1M were used. Sample labeling, hybridization, washing and drying was carried out according to the manufacturer’s instructions (Agilent Technologies). Arrays were scanned using the NimbleGen MS 200 Microarray Scanner (Nimblegen-Roche).

### mRNA-Sequencing.

Total RNA was extracted from cultured human and mouse cells using an RNeasy Mini Kit (Qiagen) and included an on-column DNase treatment to eliminate contaminating genomic DNA. Samples were analyzed on a 2100 Bioanalyzer (Agilent Technologies). All samples had an RNA Integrity Number value greater than 8. The purified RNA was used for the preparation of poly(A) selected mRNA libraries using the TruSeq RNA sample preparation kit and sequenced on an Illumina HiSEq. 4000 sequence analyzer as paired-end 76-bp reads.

### RNAPII ChIP-Seq.

ChIP-seq against RNAPII was performed as described previously (33). ChIP-derived DNA fragments were submitted for further manipulation by standard ChIP-seq library preparation techniques (Illumina) and advanced sequencing on an Illumina NextSEq. 500 sequence analyzer as 75-bp single-end reads.

## Supplementary Material

Supplementary File

Supplementary File

Supplementary File

Supplementary File

Supplementary File

Supplementary File

Supplementary File

Supplementary File

Supplementary File

Supplementary File

Supplementary File

## Data Availability

The microarray and sequencing data for this study have been deposited in the Gene Expression Omnibus (GEO) database, https://www.ncbi.nlm.nih.gov/geo (accession no. GSE143574). All other data are included in the main text and supporting information.
