# Supplementary material for: Integrated genome and transcriptome analyses reveal the mechanism of genome instability in ataxia with oculomotor apraxia 2
Source: Proc Natl Acad Sci U S A. 2022 Jan 18;119(4):e2114314119. doi: 10.1073/pnas.2114314119 (PMC8795503; doi:10.1073/pnas.2114314119)
Supplement: Supplementary File [file pnas.2114314119.sapp.pdf]

Supplementary information

Appendix 1

Contents:

SI Appendix 1, Figures S1 – S7

Supplementary Methods and Data Analyses

SI Appendix 1, Table S1

SI Appendix 1, Datasets S1-S8 are .xis files and are not contained within this document
SI Appendix 1, Movies 1 and 2 are .mov files and are not contained within this document

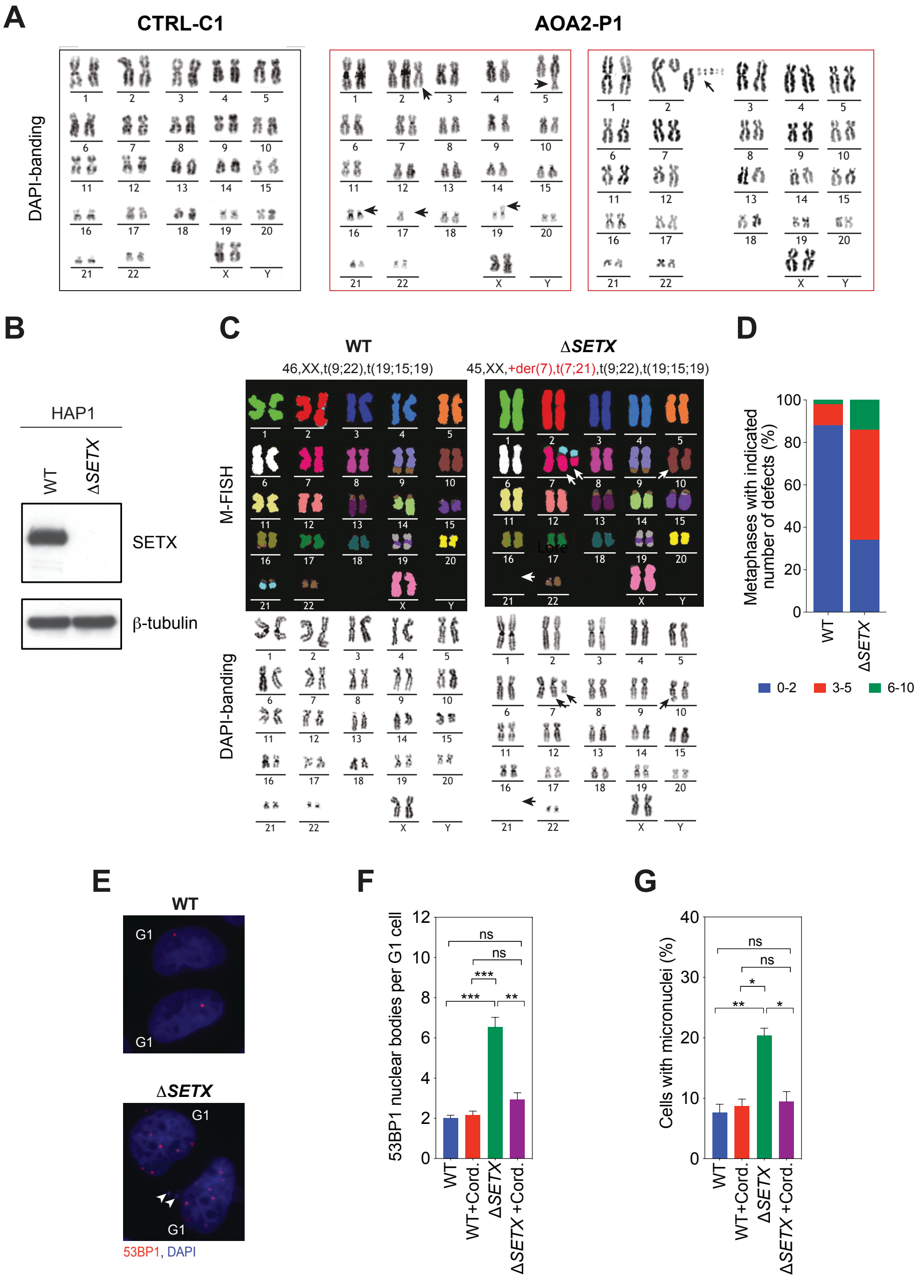

Fig. S1. SETX deficiency promotes chromosome fragility.

(A) DAP1-banding (gray-scale) images of M-FISH karyotypes shown in Fig 1F. Black arrows indicate chromosome aberrations.

(B) Western blot analysis of HAP1 WT and Δ*SETX* cells made by CRISPR/Cas9-mediated gene targeting. β-tubulin, loading control.

(C) M-FISH and DAPI-banding of metaphase spreads from HAP1 WT and Δ*SETX* cells showing deletions, translocations and chromosome fragility. Representative karyotypes are shown. Arrows indicate chromosome aberrations.

(D) Quantification of metaphases with indicated number of aberrations, as in (C). 30 metaphases were analyzed per condition.

(E) Immunostaining of HAP1 WT and Δ*SETX* cells with 53BP1 (red) antibody. Nuclear DNA was stained with DAPI (blue). Representative images of G1-phase cells are shown. White arrowheads indicate micronuclei.
(F) Quantification of 53BP1 nuclear bodies in G1 cells, as in (E). Cells were treated with or without cordycepin.
(G) Quantification of cells with indicated number of micronuclei, as in (E). Cells were treated with or without cordycepin.
Data are representative of three independent experiments. *P<0.05, **P<0.01 and ***P<0.001 by Mann-Whitney test. P≥0.05 is considered not significant (ns). Error bars represent s.e.m.

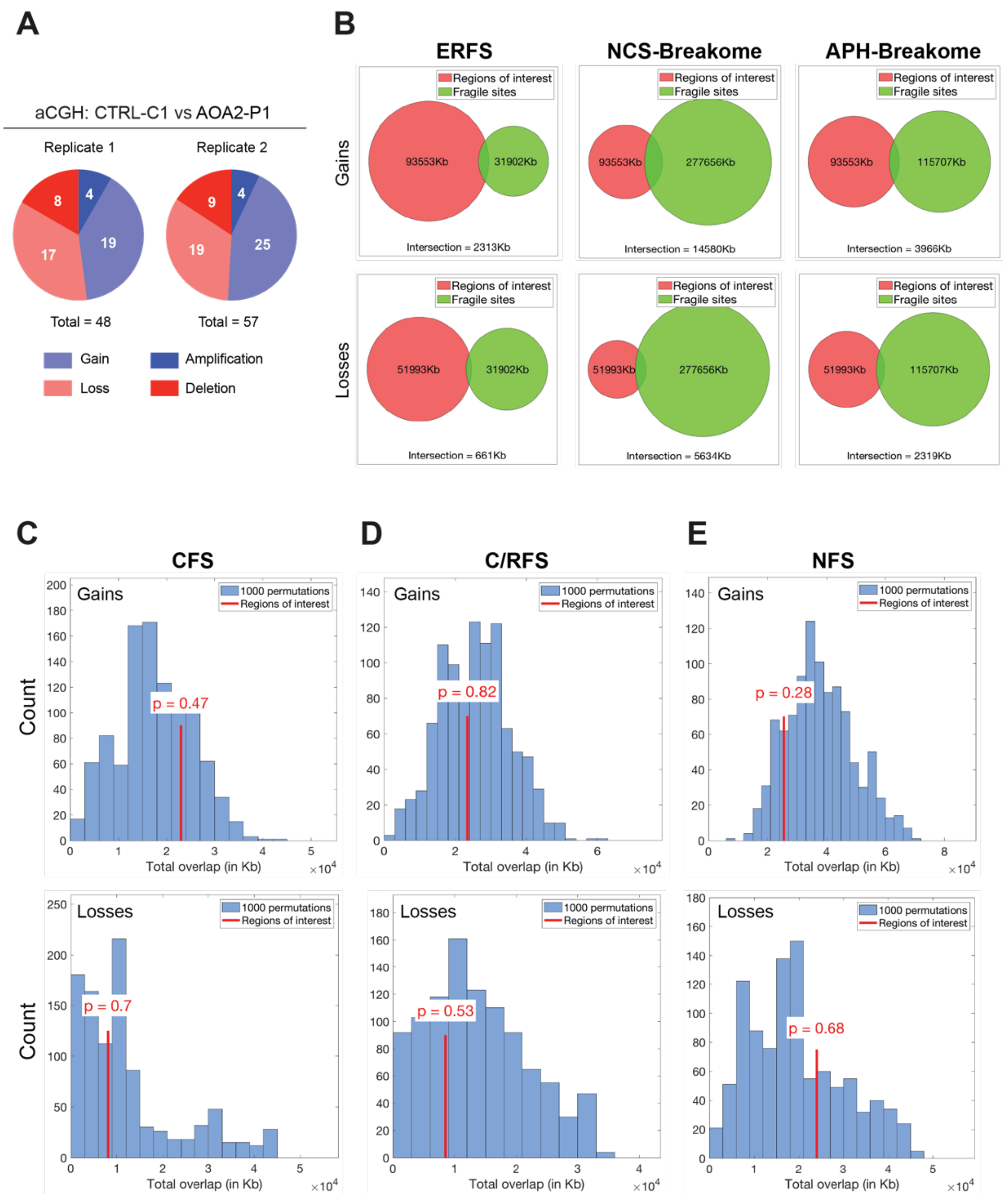

Fig. S2. Overlap analysis of CNCs identified in AOA2-P1 fibroblasts by array CGH.
(A) Pie chart representations of CNCs identified in the aCGH experiments described in Fig. 2A and B. Data from two independent experiments (replicates 1 and 2) are shown.
(B) Venn diagram of AOA2-P1 fibroblast gain or loss regions (red) and fragile sites (green) as in Fig 2C-E. Total genomic area and intersected area are reported.
(C) Top: Histogram showing overlaps between 1000 permuted AOA2-P1 fibroblast gain regions with CFSs. Bottom: Overlaps between 1000 permuted AOA2-P1 fibroblast loss regions with CFSs. Red line indicates the degree of overlap (in kb). P values for the overlap compared to permutations are indicated, with $P<0.05$ considered a significant enrichment/depletion.
(D) As (C) showing the overlaps between AOA2-P1 fibroblast gain and loss regions with the C/RFS.
(E) As (C) showing the overlaps between AOA2-P1 fibroblast gain and loss regions with the NFS.

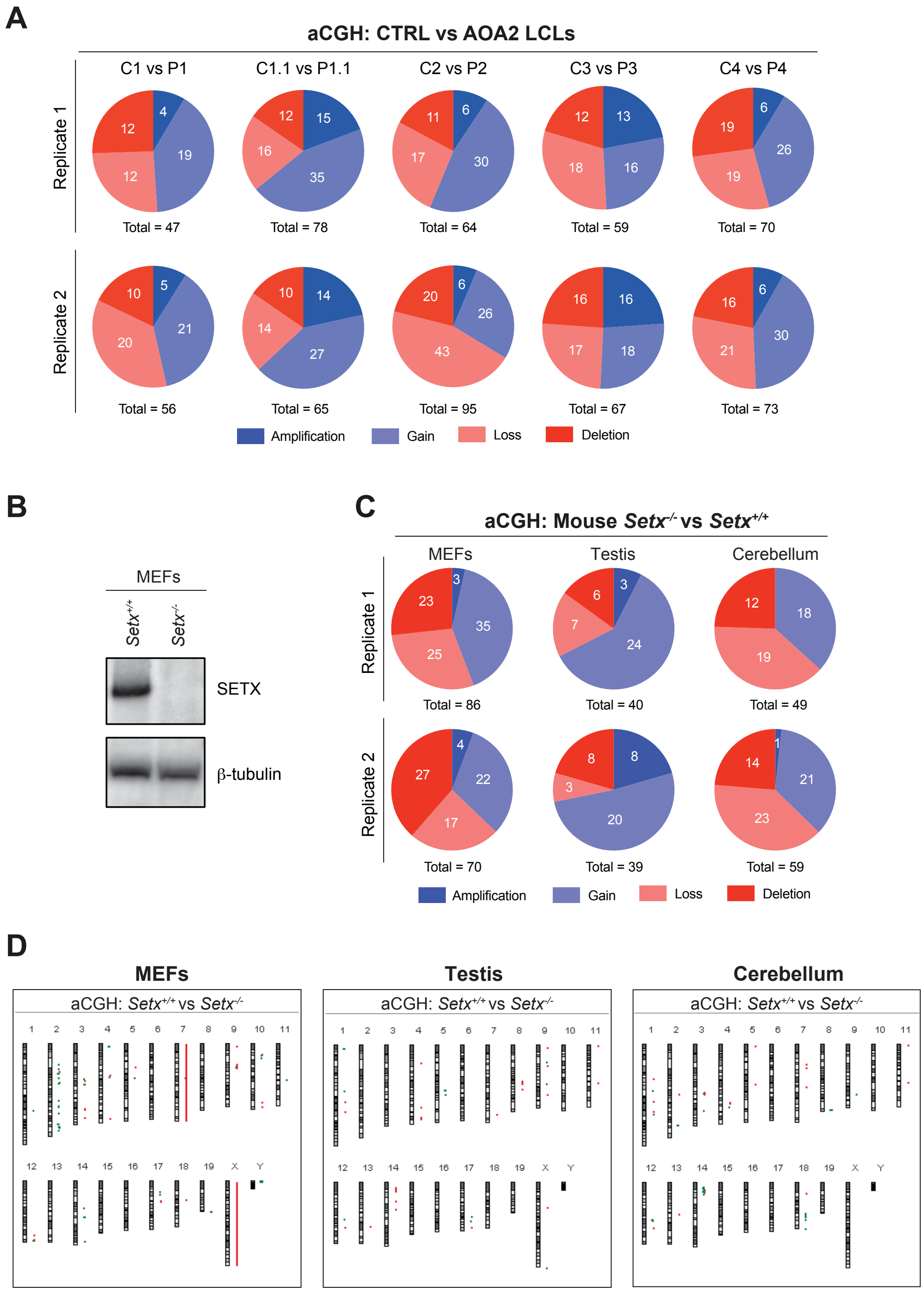

Fig. S3. Genomic aberrations associated with AOA2 LCLs and mouse *Setx* k/o.
(A) Pie chart representations of CNCs detected in two independent aCGH experiments (replicates 1 and 2) with genomic DNA from control (C1-C4 and C1.1) and AOA2 (P1-P4 and P1.1) LCLs.
(B) Western blot analysis of *Setx*$^{+/+}$ and *Setx*$^{-/-}$ MEFs. β-tubulin, loading control.
(C) As (A) but with genomic DNA from *Setx*$^{+/+}$ and *Setx*$^{-/-}$ MEFs, testis and cerebellum.
(D) Ideograms indicating the chromosomal locations of CNCs. Regions of gain and loss are indicated with red and green bars, respectively.

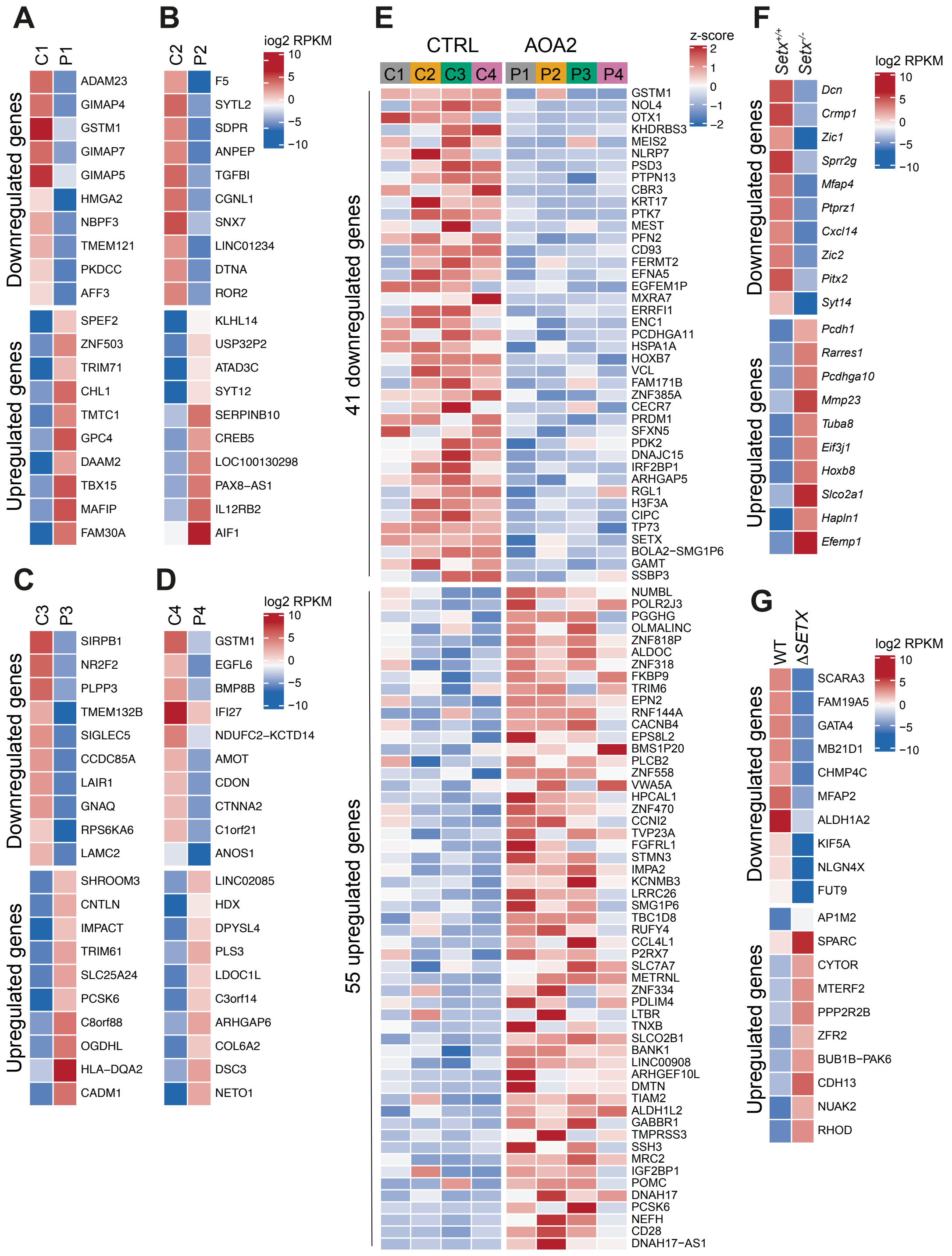

Fig. S4. Differentially expressed genes identified in *SETX*-deficient human and mouse cells.
(A) Heatmap of gene expression abundance across P1 and C1 LCLs. The top 10 most downregulated and 10 most upregulated genes, based on a fold change of normalized read count, are shown. Data are presented as log2(RPKM+0.01).
(B) As (A), using RNA-seq analyses from C2 and P2 LCLs.
(C) As (A), using RNA-seq analyses from C3 and P3 LCLs.
(D) As (A), using RNA-seq analyses from C4 and P4 LCLs.
(E) Heatmap of gene expression abundance, highlighting differences in expression between AOA2 and paired control LCLs from DESeq2 analysis (FDR≤0.05). All the downregulated (41) and upregulated (55) genes based on fold change are presented. Data are rlog transformed

to stabilize variance and normalize with respect to library size, then scaled per-gene using a z-score.

(F) As (A), using RNA-seq analyses from *Setx*$^{+/+}$ and *Setx*$^{-/-}$ mouse MEFs.

(G) As (A), using RNA-seq analyses from HAP1 WT and Δ*SETX* cells.

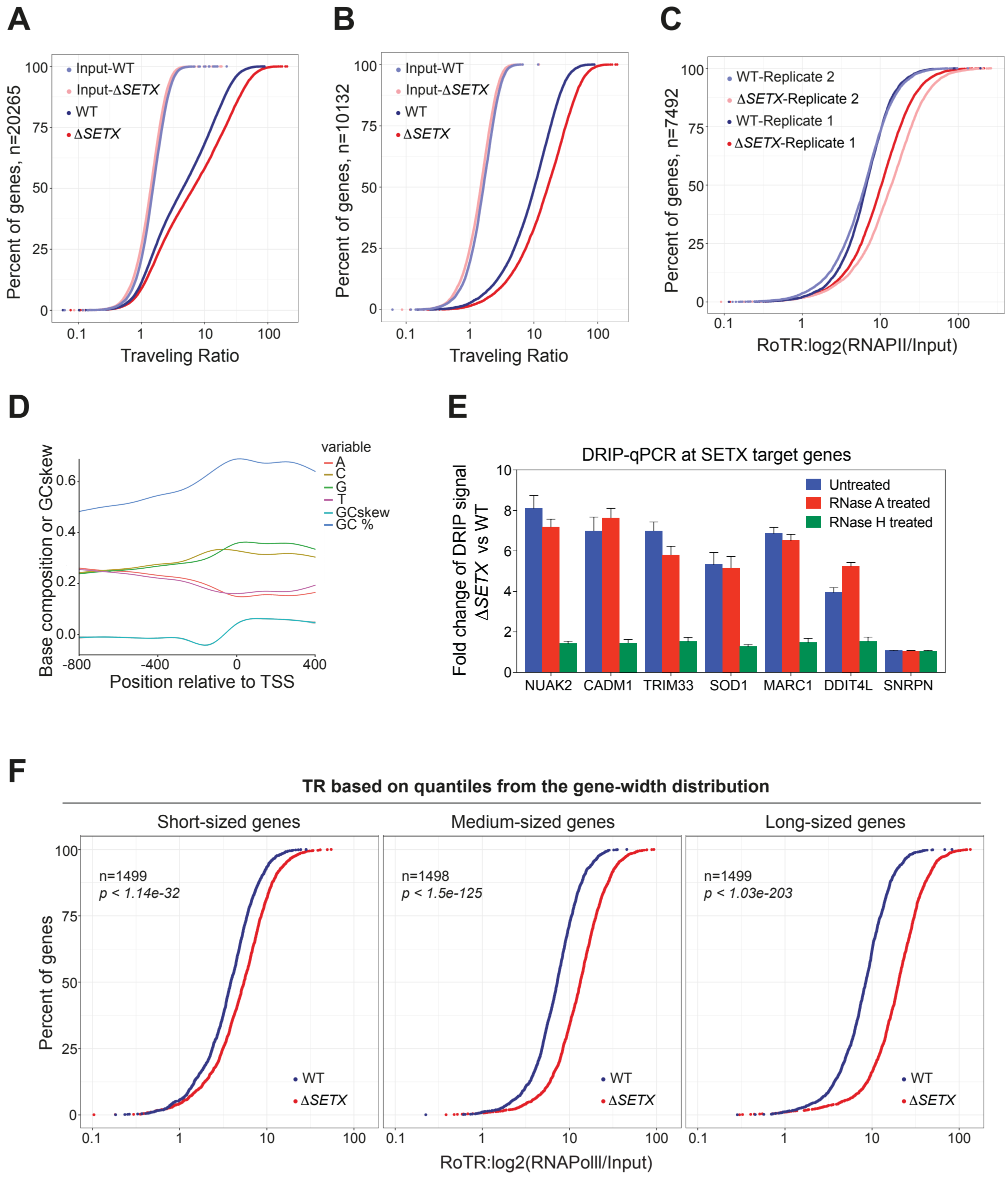

Fig. S5. Transcription stress analysis in HAP1 WT and Δ*SETX* cells.

(A) RNAPII traveling ratio (TR) distribution of all genes (n=20265) in HAP1 WT and Δ*SETX* cells, along with input controls. The y-axis indicates percent of all genes. Higher TR values indicate a higher degree of RNAPII pausing.

(B) As (A), except TR was analyzed only for genes with RNAPII peak over TSS (n=10132).

(C) As Fig 4C, except that cumulative curves of RNAPII RoTR from two independent ChIP-seq experiments are shown.

(D) Nucleotide frequencies in the [-800, +400] region around the TSS of 7492 genes identified in the RoTR analysis. GC skew and GC content (GC%) in the region are shown.

(E) DRIP-qPCR assays were carried out using HAP1 WT and Δ*SETX* cells, either untreated or treated with RNase A or RNase H. The *SETX*-target gene promoter and negative control (SNRPN) regions were analyzed. Data represent the mean ± s.e.m of three independent experiments.

(F) As Fig 4E, except that Ensembl genes are stratified into short/medium/long groupings based on the quantiles from the gene-width distributions. ‘short’ = shortest 20%, ‘medium’ = middle 40-60% of widths and ‘long’ = longest 20%.

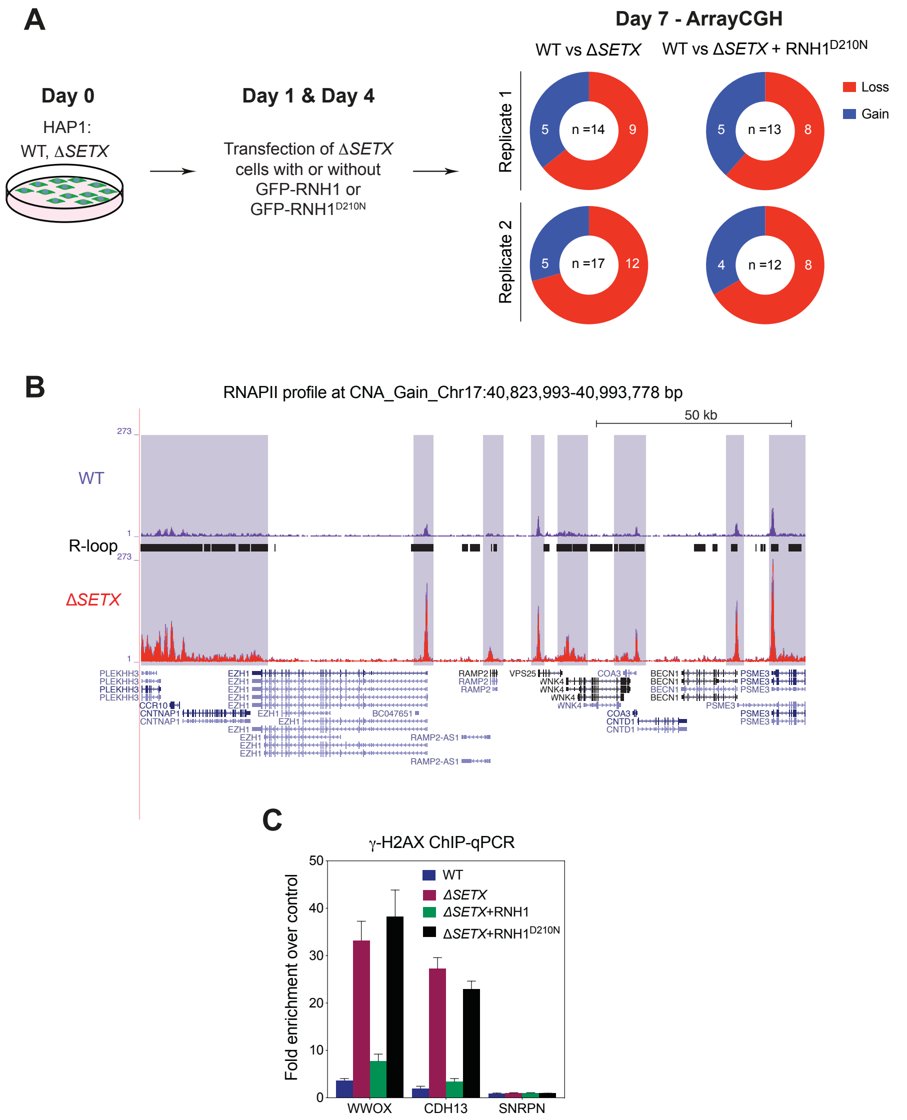

Fig. S6. Transcription stress causes chromosome instability in the absence of SETX.

(A) Schematic of aCGH experiments carried out with HAP1 WT and Δ*SETX* cells. On day 1 and day 4 post seeding, Δ*SETX* cells were transfected with or without GFP-RNaseH1 (RNH1) or GFP-RNH1^{D210N}. On day 7, aCGH was performed with WT vs WT, WT vs Δ*SETX*, WT vs Δ*SETX* + RNH1 and WT vs Δ*SETX* + RNH1^{D210N}. Donut chart representations of CNCs identified in the aCGH experiments described in Fig 5A and D. Data from two independent experiments (replicates 1 and 2) are shown.

(B) Representative genome browser screenshot of RNAPII pausing over the indicated genomic region that is amplified in Δ*SETX* (red) compared to WT (blue) cells. R-loops are shown in black. All genes in the region are shown below. Scale bar, 50kb.

(C) ChIP-qPCR analyses at *WWOX, CDH13 and SNRPN* (negative control) genes using a γ-H2AX antibody was carried out with cross-linked chromatin from HAP1 WT and Δ*SETX* cells. Fold enrichment was calculated as a ratio of γ-H2AX antibody signal versus control IgG. Data represent the mean ± s.e.m of three independent experiments.

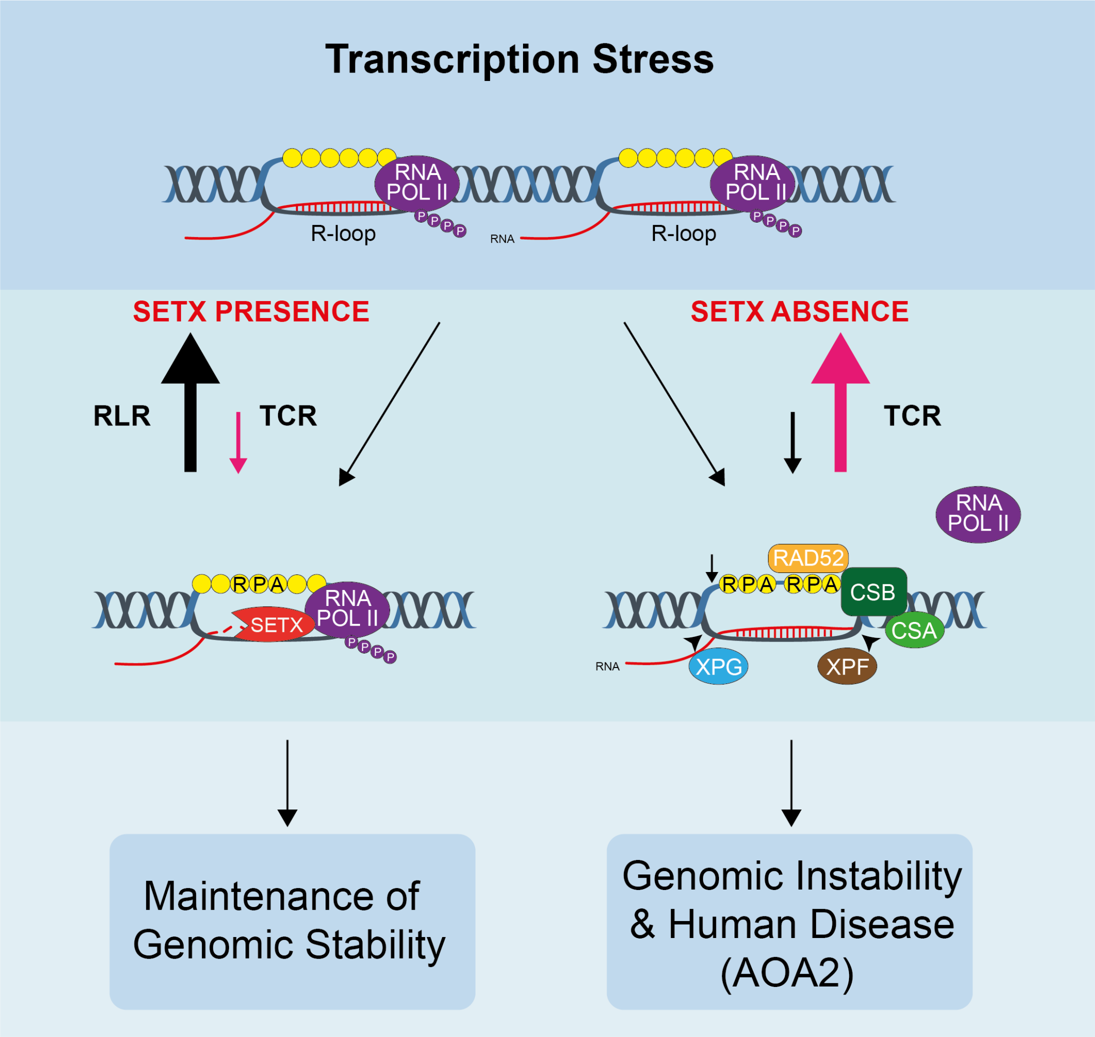

Fig. S7. Model for the role of Senataxin in response to transcription stress (RNAPII pausing, ROS damage).

Senataxin facilitates R-loop repair (RLR) by promoting the removal of R-loops at transcription sites, particularly near regions of RNAPII pausing, and thereby protects against genome stability. In the absence of SETX, CSB recognizes stalled RNAPII and transcription-coupled repair (TCR) proteins/recombination factors (e.g. RAD52) are recruited to resolve and repair transcription bubbles containing R-loops, but at the expense of faithful maintenance of the genome.

Appendix 1, **Supplementary Methods & Data Analyses**

Quantification and statistical analysis. Statistical details of experiments, including statistical tests, number of events quantified, standard deviation, standard error of the mean, and statistical significance, are reported in the figures and figure legends. GraphPad Prism7 or 8 software (GraphPad) was used for the statistical analyses of cell biology experiments.

Analysis of array comparative genomic hybridization data. Data extraction, analysis and visualization were performed using Agilent Cytogenomics 2.7.11.0 software for human samples or the Agilent Feature Extraction 12.0.0.7 and Agilent Genomic Workbench 7.0.4.0 software for mouse samples (Agilent Technologies). Analysis settings were as follows: genome, hg19 (human) or mm9 (mouse); aberration algorithm, ADM-2; threshold: 6.0; window size, 2 kb; aberration filter, ≥ 3 probes and log2ratio ≥ 0.25. The following log2ratio were used to score the aberrations: Amplification, log2ratio ≥ 2; Gain, log2ratio between 0.25 to 2; Loss, log2ratio between -0.25 to -1; Deletion, log2ratio ≤ 1.

GREAT analysis. Genomic Regions Enrichment of Annotations Tool (GREAT) was performed as described (1).

Analysis of gene expression data. Affymetrix gene expression data were analysed using Bioconductor 2.5 (https://www.bioconductor.org) running on R version 2.10.0 (2). Probeset expression measures were calculated using the Robust Multichip Average (RMA) method with the oligo package (3, 4). Differential gene expression was assessed between AOA2 and control sample groups using an empirical Bayes t-test (limma package) (5). p-values were adjusted for multiple testing correction using the Benjamini-Hochberg method (6). Probesets that exhibited an adjusted p-value of 0.05 or less were called differentially expressed. Differentially expressed probes were used to determine pathway and biological process enrichment using the Clarivate Analytics Metacore pathway analysis tool. Pathways or processes that showed FDR < 0.05 were called as enriched.

Circos plots. The Bioconductor package ggbio was used to construct the circos plots (7). The genomic locations of Aphidicolin Sensitive breakome Regions (ASR), Common Fragile Sites (CFS), Common and Rare Fragile Sites (CRFS), Early Replicating Fragile Sites (ERFS) and Neocarzinostatin Sensitive breakome Regions (NSR) were described previously (8-12). The RDC genes were described previously (13, 14).

Alignment and quantification of mRNA-seq data. Reads were aligned either against hg19 (human) or mm9 (mouse) and their respective Refseq annotations using STAR v2.5.1b (15) via the transcript quantification software RSEM v1.2.31 (16). The resulting genome alignment BAM files were sorted and indexed using SAMtools 1.3.1 (17). Duplicate reads were marked using Picard 2.1.1 (http://broadinstitute.github.io/picard). The resulting gene-level estimated read counts

were rounded to integers and further analyzed for differential expression using the Bioconductor package DESeq2 1.12.3 (18).

Differential expression of mRNA-seq data. Genes changing between AOA2 and control lymphoblastoid cell lines were tested in paired fashion. Significant genes were thresholded based on a Benjamini-Hochberg FDR ≤ 0.05, absolute fold-change ≥ 1.5 and a minimum normalized read count > 10 in either the AOA2 or control samples. Data were rlog transformed for heatmap visualization, providing variance shrinkage and normalizing with respect to library size. The heatmap showing 4 AOA2 LCLs was additionally scaled by taking gene-wise z-scores. Genes changing between human HAP1 WT and Δ*SETX* samples were assessed using an absolute fold change > 1.5, together with a normalized read count > 10 in either the Δ*SETX* or WT sample. The top 10 largest changing genes in each direction were used for heatmap visualization. Genes changing between mouse $Setx^{+/+}$ and $Setx^{-/-}$ MEFs were assessed using an absolute fold change > 2, together with a normalized read count > 30. The top 10 largest changing genes in each direction were used for heatmap visualization.

Gene Set Enrichment Analysis. Pre-ranked Gene Set Enrichment Analysis (GSEA) analysis was conducted using Bioconductor's "fgsea" package against the Gene Ontology: Biological Process gene collection defined in the Bioconductor package org.Hs.eg.db. Entrez Gene IDs were used for gene-term mappings. Gene sets containing <100 or >500 genes were discarded prior to testing. Genes not associated with an Entrez gene ID were discarded prior to testing. Genes were ranked on the Wald test statistic from the differential expression analysis. Results were thresholded using a Benjamini-Hochberg adjusted pvalue<0.05.

Overlap test. Permutation-based overlap tests were performed to assess whether human and mouse CNCs, gains and losses, were enriched for various fragile sites and/or genic regions. Gains and losses were considered independently and the analysis was restricted to the autosomes. Each gains/loss list was permuted 1000 times by assigning random genomic windows in either the hg19 (human) or mm9 (mouse) genome, with sizes equal to the original regions. These permuted windows were not permitted to overlap gaps in the reference genome, overlap each other, or overlap regions in the original gains/losses list. The permutations were then compared to each fragile site list or gene list, and the total number of overlapping base pairs determined. The number of overlapping base pairs between the true regions and the list of interest was compared to the permutation distribution by calculating a z-score and an associated two-tailed p-value. Overlap tests were also carried out to determine if gains and losses were associated with upregulated and downregulated genes. The locations of differentially regulated genes for both human and mouse were determined by comparison with the hg19 and mm9 NCBI RefSeq databases (19). For humans, 1250/1310 upregulated genes and 1046/1089 downregulated genes were identified, and for mouse, 666/684 upregulated genes and 458/470 downregulated genes. The locations were then used as the basis for overlap testing versus the gain/loss regions.

Alignment of ChIP-seq data. Single-end reads (75 bp) were aligned to the hg19 genome assembly using BWA-MEM 0.7.15 (17) with default settings. BAM files were sorted and indexed using SAMtools 1.3.1 (17). Duplicate reads were marked using Picard 2.1.1 (http://broadinstitute.github.io/picard).

Peak calling. RNAPII peaks from individual HAP1 WT and Δ*SETX* replicate samples were called against their respective input controls using MACS2 2.1.1 software (20) with the following command line options: '-g hs –q 0.05 –m 5,50'. Peak sets were thresholded for significance based on a q-value ≤ 0.01 and a fold enrichment ≥ 5, before taking the intersect of regions common to both WT or Δ*SETX* biological replicates. Peaks were further restricted to a set within +/- 500 bp of a Refseq gene's TSS.

Meta-gene profiles. Meta-gene profiles of RNAPII coverage were created using ngs.plot software (21) using standard Ensembl protein-coding gene definitions, n= 20,242. Coverage is represented as Read count Per Million mapped reads (RPM).

Traveling ratios. Travelling ratios were calculated as described (22). Briefly, each transcript was divided into: i) a promoter-proximal bin −30 bp to +300 bp around its TSS and ii) a gene body bin to the TTS. The traveling ratio is the ratio of RNAPII density in the promoter-proximal bin to that in the gene body. The most abundant transcript based on mean promoter RPKM across all samples was taken to be representative of the gene and all other transcripts were discarded. Transcripts for which a travel ratio was calculated to be zero (i.e. no reads in the promoter) or infinite (i.e., no reads in the gene body) in any of the samples were removed from the analysis, n = 20,265 transcripts (genes). An additional pre-filter, limiting the analysis to transcripts with a significant RNAPII peak (see peak calling) over their TSS leaving n = 10,132 transcripts (genes).

Ratio of traveling ratios. The ratio of traveling ratios (RoTR) was defined as the traveling ratio over a specific transcript for an RNAPII sample, divided by the traveling ratio of its respective control on the same transcript. Transcripts were limited to genes with i) a WT or Δ*SETX* RNAPII promoter peak, ii) ≥ 2kb distant from a neighboring gene on the same strand, and iii) between 2kb and 300kb in width. The most abundant transcript based on mean promoter RPKM across all samples was taken to be representative of the gene and all other transcripts were discarded. For the purposes of visualization, transcripts for which a travel ratio was calculated to be zero (i.e., no reads in promoter) or infinite (i.e., no reads in gene body) in any of the samples were removed from the analysis, leaving 7,492 transcripts (genes). Genes were stratified into width categories based on the quantiles of the gene width distribution: short: <20%, medium: 40-60% and long: >80%. A Wilcoxon rank sum test was used to assess the significance of differences between RoTR distributions.

GCskew. GCskew (strand asymmetry in the distribution of guanines and cytosines) was calculated around the TSS (+/- 400 bp) of the 7492 genes identified in the RoTR analysis as follows: GC skew = (G − C)/(G + C)

Base composition. The proportion of each base was calculated around the TSS (+/- 400bp or -800 to +400bp) of the 7492 genes identified in the RoTR analysis at single bp resolution. GC-content was calculated as the percentage of the nucleotides that possess either “G” or “C” bases. GCskew was calculated as described. Where appropriate a loess curve was fitted to the data.

BigWig files. BigWig files representing genome-wide read depth coverage were generated from BAM alignment files using BEDtools’ genomeCoverageBed function (23). BedGraph files were in turn converted to bigWig format using the bedGraphToBigWig function from the KentTools package (24). Genome browser profiles were generated using the UCSC browser.

SI Appendix, Table S1

Reagent/Resource	Reference or Source	Identifier or Catalog Number
**Experimental models**		
Human: AOA2-P1 Fibroblast (also known as SETX-1RM)	AOA2 patient fibroblast with homozygous *SETX* mutation, c.6106+3393_7101-22del20648ins25	(25)
Human: AOA2-P1 LCL (LCL related to SETX-1RM)	AOA2 patient LCL with homozygous *SETX* mutation, c.6106+3393_7101-22del20648ins25	(26)
Human: AOA2-P1.1 LCL (also known as SETX-2RM)	AOA2 patient LCL with homozygous *SETX* mutation, c.7199+5G>A	(25, 26)
Human: AOA2-P2 LCL (Also known as P2062)	AOA2 patient LCL with compound heterozygous *SETX* mutation, c.7240C>T; c.7626delG	(27, 28)
Human: AOA2-P3 LCL (also known as IT1)	AOA2 patient LCL with homozygous *SETX* mutation, c.3466delG	(26, 29)
Human: AOA2-P4 LCL (also known as EM594)	AOA2 patient LCL with homozygous *SETX* mutation, c.1669C>T	(30)
Human: CTRL-C1 Fibroblast (also known as NFF)	Control for AOA2-P1 fibroblast	(25, 26)
Human: CTRL-C1 LCL	Age- and sex-matched control for AOA2-P1 LCL	(25, 26)
Human: CTRL-C1.1 LCL	Age- and sex-matched control for AOA2-P1.1 LCL	(25, 26)
Human: CTRL-C2 LCL	Age- and sex-matched control for AOA2-P2 LCL	(27, 28)
Human: CTRL-C3 LCL	Age- and sex-matched control for AOA2-P3 LCL	(26, 29)
Human: CTRL-C4 LCL (also known as CR26)	Age- and sex-matched control for AOA2-P4 LCL	(30)
Human: CS (also known as $CS1AN_{SV}$)	Jesper Svejstrup Lab	(31)
Human: CS+CSB	Jesper Svejstrup Lab	(31)
Human: HAP1-WT	Horizon Discovery	(32)
Human: HAP1-Δ*SETX*	This paper	N/A
Human: MRC5	ATCC	ATCC CCL-171
Human: MRC5 VA (SV40-transformed)	The Francis Crick Institute Cell Services	N/A
Human: U2OS-GFP-m53BP1	Jiri Lukas Lab	(33)
Mouse: C57BL/6J	The Jackson Laboratory	Cat#000664
Mouse: *Setx*$^{-/-}$ knock out in C57BL/6J	Martin Lavin Lab	(34)

Mouse: Mouse Embryonic Fibroblasts *Setx*$^{+/+}$	This paper	(34)
Mouse: Mouse Embryonic Fibroblasts *Setx*$^{-/-}$	This paper	(34)
**Recombinant DNA**		
pEGFP-hM27RNaseH1	Robert Crouch Lab	(35)
pEGFP-hM27RNaseH1^{D210N}	This paper	N/A
pSUPER.puro	OligoEngine	Cat#VEC-PBS-0008
pX330	Addgene	Cat#42230
pX330-sgRNA-*SETX*	This paper	N/A
**Antibodies**		
Rabbit polyclonal anti-53BP1	Abcam	Cat#ab36823; RRID: AB_722497
Rabbit polyclonal anti-53BP1	Novus Biologicals	Cat#NB100-304; RRID: AB_10003037
Rabbit polyclonal anti-GFP	Abcam	Cat#ab290; RRID: AB_303395
Rabbit polyclonal anti-γH2AX phospho S139	Abcam	Cat#ab2893; RRID: AB_303388
Rabbit polyclonal anti-RPA	Abcam	Cat#ab10359; RRID: AB_297095
Rabbit polyclonal anti-SETX	Merck Millipore	Cat#ABN421; RRID: AB_2819063
Rabbit polyclonal anti-SETX	Novus Biologicals	Cat#NBP1-94712; RRID: AB_11010824
Rabbit polyclonal anti-XPF	Santa Cruz	Cat#sc-28718; RRID: AB_2277828
Rabbit polyclonal anti-CSB	Bethyl Laboratories	Cat#A301-345A; RRID: AB_937843
Mouse monoclonal anti-cyclin A	Santa Cruz	Cat#sc-56299; RRID: AB_782328
Mouse monoclonal anti-RAD52	Santa Cruz	Cat#sc-365341; RRID: AB_10851346
Mouse monoclonal anti-RNAPII CTD clone 4H8	The Francis Crick Institute	N/A
Mouse monoclonal anti-RNA/DNA hybrids (S9.6)	Stephen Leppla lab and the Francis Crick Institute	(36)
Mouse monoclonal anti-β-tubulin	Santa Cruz	Cat#sc-5274; RRID: AB_2288090
Mouse monoclonal anti-XPG clone 8H7	The Francis Crick Institute	N/A
Rabbit IgG, polyclonal - negative control	Abcam	Cat#ab171870; RRID: AB_2687657
Mouse IgG, polyclonal - negative control	Diagenode	Cat#C15400001; RRID: AB_2722553

Bridging Antibody for Mouse IgG	Active Motif	Cat#53017
Goat anti-mouse Immunoglobulins/HRP	Agilent	Cat#P0447; RRID: AB_2617137
Goat anti-rabbit Immunoglobulins/HRP	Agilent	Cat#P0448; RRID: AB_2617138
Alexa Fluor 488 Goat anti-Rabbit IgG (H+L)	ThermoFisher	Cat#A-11070; RRID: AB_2534114
Alexa Fluor 568 Donkey anti-Mouse IgG (H+L)	ThermoFisher	Cat#A-10037; RRID: AB_2534013
**Oligonucleotides and other sequence-based reagents**		
Primer for ChIP-qPCR: ATP5C1-TSS-Forward: GGCTGTGGCTACCATGTTCT	Sigma-Aldrich	(37)
Primer for ChIP-qPCR: ATP5C1-TSS-Reverse: AAGCTCTTCCATCCCTTGC	Sigma-Aldrich	(37)
Primer for ChIP-qPCR: ATP5G1-TSS-Forward: GAGACCAAGGGCTAAAGCTG	Sigma-Aldrich	(37)
Primer for ChIP-qPCR: ATP5G1-TSS-Reverse: GGGAAATACCCCTCCACACT	Sigma-Aldrich	(37)
Primer for ChIP-qPCR: ATRAID-TSS-Forward: ACCAAGGGAACGGAAAATG	Sigma-Aldrich	(37)
Primer for ChIP-qPCR: ATRAID-TSS-Reverse: CCGACCTCAAACTTGCTTCT	Sigma-Aldrich	(37)
Primer for ChIP-qPCR: CLSPN-TSS-Forward: GGCTGAGGGAATCAGAGACA	Sigma-Aldrich	(37)
Primer for ChIP-qPCR: CLSPN-TSS-Reverse: GGGCGTGTGCATAAACTCA	Sigma-Aldrich	(37)
Primer for ChIP-qPCR: COL4A3BP-TSS-Forward: CCGGATTTTCTCTTCCCTTC	Sigma-Aldrich	(37)
Primer for ChIP-qPCR: COL4A3BP-TSS-Reverse: GAGCGGTGAAGGAAGCCTAC	Sigma-Aldrich	(37)
Primer for ChIP-qPCR: GADD45A-TSS-Forward: GCCTGTGAGTGAGTGCAGAA	Sigma-Aldrich	(37)
Primer for ChIP-qPCR: GADD45A-TSS-Reverse: CGACTCACCTTTCGGTCTTC	Sigma-Aldrich	(37)
Primer for ChIP-qPCR: LENG8-TSS-Forward: CGCACTTACGCATGAACATT	Sigma-Aldrich	(37)
Primer for ChIP-qPCR: LENG8-TSS-Reverse: AGACTCCGTCTCCGAGAACA	Sigma-Aldrich	(37)

Primer for ChIP-qPCR: PIGH-TSS-Forward: CGCTACTACTCCCCGTCCT	Sigma-Aldrich	(37)
Primer for ChIP-qPCR: PIGH-TSS-Reverse: CTCGCAGAGGGTGAAGAGTC	Sigma-Aldrich	(37)
Primer for ChIP-qPCR: PMPCA-TSS-Forward: AGCGGAAGTGACGACTGAAG	Sigma-Aldrich	(37)
Primer for ChIP-qPCR: PMPCA-TSS-Reverse: ACGGAGCGTCCAGAAAGACT	Sigma-Aldrich	(37)
Primer for ChIP-qPCR: PMS2-TSS-Forward: AGCTGAGAGCTCGAGGTGAG	Sigma-Aldrich	(37)
Primer for ChIP-qPCR: PMS2-TSS-Reverse: GAGATCGCTGCAACACTGAG	Sigma-Aldrich	(37)
Primer for ChIP-qPCR: SMG5-TSS-Forward: CCACACTAAGCGGCTTTACC	Sigma-Aldrich	(37)
Primer for ChIP-qPCR: SMG5-TSS-Reverse: AAAATTGCACCCTCCTTCCT	Sigma-Aldrich	(37)
Primer for ChIP-qPCR: SNRPD2-TSS-Forward: GGCAGATGTTTTAGGGATGC	Sigma-Aldrich	(37)
Primer for ChIP-qPCR: SNRPD2-TSS-Reverse: CGCCTTGGCCTACTAAAGTG	Sigma-Aldrich	(37)
Primer for ChIP-qPCR: SNRPN-Negative Control-Forward: GCCAAATGAGTGAGGATGGT	Sigma-Aldrich	(38)
Primer for ChIP-qPCR: SNRPN-Negative Control-Reverse: TCCTCTCTGCCTGACTCCAT	Sigma-Aldrich	(38)
Primer for ChIP-qPCR: THOP1-TSS-Forward: CGCAGGTACCGACTACCC	Sigma-Aldrich	(37)
Primer for ChIP-qPCR: THOP1-TSS-Reverse: ATTCATTCGGCAAACGAGAG	Sigma-Aldrich	(37)
Primer for mutageneis: hM27RNaseH1-D210N-Forward: CTGGTTCTGTATACAAACAGTATGTTTACGA	This paper	N/A
Primer for mutageneis: hM27RNaseH1-D210N-Reverse: TCGTAAACATACTGTTTGTATACAGAACCAG	This paper	N/A
sgRNA oligonucleotide targeting SETX: Guide_Top: CACCGCGTTCATGTAGAAGCAAGTA	This paper	N/A
sgRNA oligonucleotide targeting SETX: Guide_Bottom:	This paper	N/A

AAACTACTTGCTTCTACATGA ACGC		
siRNA: ON-TARGETplus Human SETX siRNA UAGCACAGGUUGUUAAUCA	Dharmacon, Horizon Discovery	Cat#J-021420-08-0005
siRNA: ON-TARGETplus Non-targeting Control siRNA #1	Dharmacon, Horizon Discovery	Cat#D-001810-01-05
**Chemicals, enzymes and other reagents**		
Acetic acid	ThermoFisher	Cat#10304980
Acetone	ThermoFisher	Cat#10324930
Acetonitrile	Sigma-Aldrich	Cat#271004
Aminoallyl-dUTP-XX-ATTO-488	Stratech	Cat#NU-803-XX-488-L-JEN
Aminoallyl-dUTP-ATTO-425	Stratech	Cat# NU-803-425-L-JEN
Aminoallyl-dUTP-Cy3	Stratech	Cat#NU-803-CY3-L-JEN
Aminoallyl-dUTP-CY5	Stratech	Cat#NU-803-CY5-L-JEN
Aminoallyl-dUTP-Texas Red	Stratech	Cat#NU-803-TXR-L-JEN
Ampicillin sodium salt	Sigma-Aldrich	Cat#A0166
Colcemid	Roche	Cat#10295892001
cOmplete, Mini, EDTA-free Protease Inhibitor Cocktail	Roche	Cat#11836170001
Cordycepin 5′-triphosphate sodium salt	Sigma-Aldrich	Cat#C9137
Cot-I Human DNA	Agilent	Cat#5190-3393
Cot-I Mouse DNA	ThermoFisher	Cat#18440016
Cytochalasin B	Sigma-Aldrich	Cat#C6762
Deoxynucleotide (dNTP) Solution Mix	NEB	Cat# N0447L
Dextran Sulfate	Merck Millipore	Cat#S4030
DNase I, RNase-free	Roche	Cat#4716728001
Ethanol	ThermoFisher	Cat#10048291
Ethanol (absolute)	Sigma-Aldrich	Cat#E7023
Fetal Bovine Serum	Sigma-Aldrich	Cat#F2442
Fetal Bovine Serum	Sigma-Aldrich	Cat#F7524
Formaldehyde solution	Sigma-Aldrich	Cat#F8775
Formamide	Sigma-Aldrich	Cat#47671
Gibco DMEM medium	ThermoFisher	Cat#11995073
Gibco DMEM, no phenol red	ThermoFisher	Cat#21063029
Gibco IMDM medium	ThermoFisher	Cat#21980065
Gibco Opti-MEM Medium	ThermoFisher	Cat#31985062
Gibco RPMI 1640 Medium	ThermoFisher	Cat#21875034
Glycine	Sigma-Aldrich	Cat#G8898
Hydrogen peroxide solution	Sigma-Aldrich	Cat#H1009

Immobilon-P PVDF Membrane	Merck Millipore	Cat# IPVH00010
Invitrogen NuPAGE 3-8% Tris-Acetate Protein Gels	ThermoFisher	Cat#EA0378BOX
Invitrogen NuPAGE 4-12% Bis-Tris Protein Gels	ThermoFisher	Cat# NP0321BOX
iQ SYBR Green Supermix	Bio-Rad	Cat#170-8884
Kanamycin Monosulphate	Formedium	Cat#KAN0025
L-Glutamine	ThermoFisher	Cat#25030024
Methanol	ThermoFisher	Cat#10396090
Penicillin-Streptomycin	Sigma-Aldrich	Cat#P4333
Phenol:Chloroform, pH 4.5	ThermoFisher	Cat#AM9722
PhosSTOP	Roche	Cat#4906837001
Pierce 16% Formaldehyde, Methanol-free	ThermoFisher	Cat#28908
ProLong Gold Antifade Mountant with DAPI	ThermoFisher	Cat#P36935
Propidium Iodide	Sigma-Aldrich	Cat#P4864
Protein G Magnetic Beads	Active Motif	Cat#53033
Proteinase K	Sigma-Aldrich	Cat#P4850
Restriction Enzymes	NEB	https://www.neb.uk.com/
RNase A	Qiagen	Cat#19101
RNase A	ThermoFisher	Cat#EN0531
RNase H	NEB	Cat#M0297L
SlowFade Gold Antifade Mountant with DAPI	ThermoFisher	Cat#S36939
Stabilization and Drying Solution	Agilent	Cat#5185-5979
**Software**		
Adobe Illustrator CC	Adobe	https://www.adobe.com/uk/products/illustrator.html
Adobe Photoshop CC	Adobe	https://www.adobe.com/uk/products/photoshop.html
Agilent Cytogenomics 2.7.11.0	Agilent	https://www.agilent.com/en/genomics-software-downloads
Agilent Feature Extraction 12.0.0.7	Agilent	https://www.agilent.com/en/genomics-software-downloads
Agilent Genomic Workbench 7.0.4.0	Agilent	https://www.agilent.com/en/genomics-software-downloads
BEDtools	(23)	https://bedtools.readthedocs.io/en/latest/
BWA-MEM v0.7.15	(17)	http://bio-bwa.sourceforge.net/

DAVID Bioinformatics Resources	(39)	https://david.ncifcrf.gov/
DESeq2 v1.12.3	(18)	https://bioconductor.org/packages/release/bioc/html/DESeq2.html
FlowJo v9.9	Tree Star, Inc.	https://www.flowjo.com/solutions/flowjo/downloads
Ggbio 1.20.1	(7)	https://bioconductor.org/packages/release/bioc/html/ggbio.html
GREAT v4.0.4	(1)	http://great.stanford.edu/
IDEAS v6.2	Amnis Corporation	N/A
ImageJ32	NIH	https://imagej.nih.gov/ij/
INSPIRE	Amnis Corporation	N/A
KentTools	(24)	http://hgdownload.soe.ucsc.edu/admin/exe/
MACS2 v2.1.1	(20)	https://github.com/taoliu/MACS
MiSigDB	(40, 41)	http://www.broadinstitute.org/gsea/msigdb/
Ngs.plot	(21)	https://github.com/shenlab-sinai/ngsplot
Picard v2.1.1	Broad Institute	http://broadinstitute.github.io/picard
R/Bioconductor	Bioconductor	https://www.bioconductor.org/
RSEM v1.2.31	(16)	https://github.com/deweylab/RSEM
SAMtools v1.3.1	(17)	http://samtools.sourceforge.net/
SimplePCI 6 Imaging Software	Hamamatsu Corporation	N/A
SmartCapture software	Digital Scientific	http://dsuk.biz/DSUK/Downloads.html
SmartType Karyotyper Software	Digital Scientific	http://dsuk.biz/DSUK/Downloads.html
STAR v2.5.1b	(15)	https://github.com/alexdobin/STAR
UCSC Genome Browser	University of California	https://genome.ucsc.edu/
Volocity v6.3	PerkinElmer	http://cellularimaging.perkinelmer.com/downloads/details.php?id=14
**Other**		
ChIP-IT Express Kit	Active Motif	Cat#53008
Chromatin IP DNA Purification Kit	Active Motif	Cat#58002
DNeasy Blood & Tissue Kit	Qiagen	Cat#69506

FL-Ovation cDNA Biotin Module v2	NuGEN	Cat#4200-60
GeneChip Human Gene 1.0 ST Array	Affymetrix	Cat#901087
GenomePlex Complete Whole Genome Amplification Kit	Sigma-Aldrich	Cat# WGA2
GenomePlex WGA Reamplification Kit	Sigma-Aldrich	Cat# WGA3
Hard-Shell Thin-Wall 96-Well Skirted PCR Plates	Bio-Rad	Cat#HSP-9655
Hybridization Chamber Kit - SureHyb enabled, Stainless	Agilent	Cat#G2534A
Hybridization Gasket Slide Kit	Agilent	Cat#G2534-60002
Invitrogen Lipofectamine 2000	ThermoFisher	Cat#11668019
Invitrogen Lipofectamine RNAiMAX	ThermoFisher	Cat#13778150
Microseal 'B' Adhesive Seals	Bio-Rad	Cat#MSB-1001
Nunc Lab-Tek Chambered Coverglass	ThermoFisher	Cat#155380PK
Oligo aCGH/ChIP-on-chip Hybridization Kit	Agilent	Cat#5188-5220
Oligo aCGH/ChIP-on-chip Wash Buffer Kit	Agilent	Cat#5188-5226
Quikchange Site-Directed Mutagenesis Kit	Agilent	Cat#210513
RNeasy Mini Kit	Qiagen	Cat#74104
SurePrint G3 Human CGH 2x400K Microarray	Agilent	Cat#G4448A
SurePrint G3 Mouse CGH 1x1M Microarray	Agilent	Cat#G4838A
SureTag Complete DNA Labeling Kit	Agilent	Cat#5190-4240
TruSeq Stranded mRNA Library Prep Kit	Illumina	Cat#20020595
QIAamp DNA Mini Kit	Qiagen	Cat#51304
QIAfilter Plasmid Midi Kit	Qiagen	Cat#12243
QIAprep Spin Miniprep Kit	Qiagen	Cat#27106
QIAquick PCR Purification Kit	Qiagen	Cat#28106
WT-Ovation Pico RNA Amplification system	NuGen	Cat#3300-12

References

1. McLean CY, *et al.* (2010) GREAT improves functional interpretation of cis-regulatory regions. *Nat. Biotechnol.* 28:495-501.
2. Team RC (2008) R: A language and environment for statistical computing. in *R Foundation for Statistical Computing*.
3. Carvalho BS & Irizarry RA (2010) A framework for oligonucleotide microarray preprocessing. *Bioinformatics* 26:2363-2367.
4. Gautier L, Cope L, Bolstad BM, & Irizarry RA (2004) Affy-analysis of Affymetrix GeneChip data at the probe level. *Bioinformatics* 20:307-315.
5. Smyth GK (2005) limma: Linear models for microarray data. *Bioinformatics and Computational Biology Soulutions Using R and Bioconductor. Statistics for Biology and Health*, eds Gentleman RC, Carey VJ, Huber W, & Irizarry RA (Springer, New York), pp 397-420.
6. Benjamini Y & Hochberg Y (1995) Controlling the false discovery rate: A practical and powerful approach to multiple testing. *J. Royal. Stat. Soc. Series B* 57:289-300.
7. Yin T, Cook D, & Lawrence M (2012) ggbio: an R package for extending the grammar of graphics for genomic data. *Genome Biol.* 13:R77.
8. Helmrich A, Stout-Weider K, Hermann K, Schrock E, & Heiden T (2006) Common fragile sites are conserved features of human and mouse chromosomes and relate to large active genes. *Genome Res.* 16:1222-1230.
9. Bignell GR, *et al.* (2010) Signatures of mutation and selection in the cancer genome. *Nature* 463:893-898.
10. Fungtammasan A, Walsh E, Chiaromonte F, Eckert KA, & Makova KD (2012) A genome-wide analysis of common fragile sites: what features determine chromosomal instability in the human genome? *Genome Res.* 22:993-1005.
11. Barlow JH, *et al.* (2013) Identification of early replicating fragile sites that contribute to genome instability. *Cell* 152:620-632.
12. Crosetto N, *et al.* (2013) Nucleotide-resolution DNA double-strand break mapping by next-generation sequencing. *Nat. Methods* 10:361-365.
13. Wei PC, *et al.* (2016) Long neural genes harbor recurrent DNA break clusters in neural stem/progenitor cells. *Cell* 164:644-655.
14. Wei PC, *et al.* (2018) Three classes of recurrent DNA break clusters in brain progenitors identified by 3D proximity-based break joining assay. *Proc. Natl. Acad. Sci .U. S. A.* 115:1919-1924.
15. Dobin A, *et al.* (2013) STAR: ultrafast universal RNA-seq aligner. *Bioinformatics* 29:15-21.
16. Li B & Dewey CN (2011) RSEM: accurate transcript quantification from RNA-Seq data with or without a reference genome. *BMC Bioinformatics* 12:323.
17. Li H, *et al.* (2009) The sequence alignment/map format and SAMtools. *Bioinformatics* 25:2078-2079.
18. Love MI, Huber W, & Anders S (2014) Moderated estimation of fold change and dispersion for RNA-seq data with DESeq2. *Genome Biol.* 15:550.
19. Pruitt KD, *et al.* (2014) RefSeq: an update on mammalian reference sequences. *Nucleic Acids Res.* 42:D756-D763.
20. Zhang Y, *et al.* (2008) Model-based analysis of ChIP-Seq (MACS). *Genome Biol.* 9:R137.
21. Shen L, Shao N, Liu X, & Nestler E (2014) ngs.plot: Quick mining and visualization of next-generation sequencing data by integrating genomic databases. *BMC Genomics* 15:284.
22. Rahl PB, *et al.* (2010) c-Myc regulates transcriptional pause release. *Cell* 141:432-445.
23. Quinlan AR & Hall IM (2010) BEDTools: a flexible suite of utilities for comparing genomic features. *Bioinformatics* 26:841-842.

24. Kent WJ, Zweig AS, Barber G, Hinrichs AS, & Karolchik D (2010) BigWig and BigBed: enabling browsing of large distributed datasets. *Bioinformatics* 26:2204-2207.

25. Suraweera A, *et al.* (2007) Senataxin, defective in ataxia oculomotor apraxia 2, is involved in the defence against oxidative DNA damage. *J. Cell. Biol.* 177:969-979.

26. Criscuolo C, *et al.* (2006) Ataxia with oculomotor apraxia type 2: a clinical, pathologic, and genetic study. *Neurology* 66:1207-1210.

27. Anheim M, *et al.* (2009) Ataxia with oculomotor apraxia type 2: clinical, biological and genotype/phenotype correlation study of a cohort of 90 patients. *Brain* 132:2688-2698.

28. Nanetti L, *et al.* (2013) SETX mutations are a frequent genetic cause of juvenile and adult onset cerebellar ataxia with neuropathy and elevated serum alpha-fetoprotein. *Orphanet. J. Rare Dis.* 8:123.

29. Fogel BL, *et al.* (2014) Mutation of Senataxin alters disease-specific transcriptional networks in patients with ataxia with oculomotor apraxia type 2. *Hum. Mol. Genet.* 23:4758-4769.

30. Airoldi G, *et al.* (2010) Characterization of two novel SETX mutations in AOA2 patients reveals aspects of the pathophysiological role of senataxin. *Neurogenetics* 11:91-100.

31. Wang Y, *et al.* (2014) Dysregulation of gene expression as a cause of Cockayne syndrome neurological disease. *Proc. Natl. Acad. Sci .U. S. A.* 111:14454-14459.

32. Carette JE, *et al.* (2011) Ebola virus entry requires the cholesterol transporter Niemann-Pick C1. *Nature* 477:340-343.

33. Bekker-Jensen S, Lukas C, Melander F, Bartek J, & Lukas J (2005) Dynamic assembly and sustained retention of 53BP1 at the sites of DNA damage are controlled by Mdc1/NFBD1. *J. Cell Biol.* 170:201-211.

34. Becherel OJ, *et al.* (2013) Senataxin plays an essential role with DNA damage response proteins in meiotic recombination and gene silencing. *PLoS Genet.* 9:e1003435.

35. Suzuki Y, *et al.* (2010) An upstream open reading frame and the context of the two AUG codons affect the abundance of mitochondrial and nuclear RNase H1. *Mol. Cell. Biol.* 30:5123-5134.

36. Hu Z, Zhang A, Storz G, Gottesman S, & Leppla SH (2006) An antibody-based microarray assay for small RNA detection. *Nucleic Acids Res* 34:e52.

37. Chen L, *et al.* (2017) R-ChIP using inactive RNase H reveals dynamic coupling of R-loops with transcriptional pausing at gene promoters. *Mol. Cell* 68:745-757.

38. Sanz LA, *et al.* (2016) Prevalent, dynamic and conserved R-loop structures associate with specific epigenomic signatures in mammals. *Mol. Cell* 63:167-178.

39. Huang DW & Lempicki RA (2009) Systematic and integrative analysis of large gene lists using DAVID bioinformatics resources. *Nat. Protoc.* 4:44-57.

40. Liberzon A, *et al.* (2011) Molecular signatures database (MSigDB) 3.0. *Bioinformatics* 27:1739-1740.

41. Subramanian A, *et al.* (2005) Gene set enrichment analysis: a knowledge-based approach for interpreting genome-wide expression profiles. *Proc. Natl. Acad. Sci. U. S. A.* 102:15545-15550.
